# AI-driven adaptive training for utility employees: a three-wave cluster-randomized field experiment on cognitive-load and personalization-fit pathways

**DOI:** 10.3389/fpsyg.2026.1944626

**Published:** 2026-09-17

**Authors:** Cuilian Tang, Yaxu Cao, Quanyu Shen, Shaohua Kang, Yanan Yang, Pengcheng Chang, Mengmeng Fan

**Affiliations:** 1 State Grid Zhengzhou Power Supply Company, Zhengzhou, China; 2 School of Management, Zhengzhou University, Zhengzhou, China

**Keywords:** adaptive learning, cognitive load, field experiment, knowledge graph, personalization, self-efficacy, training transfer, workplace training

## Abstract

Evidence that AI-driven adaptive learning outperforms uniform instruction comes almost entirely from student samples in educational settings; whether, why, and for whom it works in real-world workplace training remains largely untested. We conducted a three-wave, matched-pair, cluster-randomized field experiment across six county-level power supply companies of a Chinese provincial electric power utility. Employees (*N* = 3,000 invited; analysis sample *N* = 2,574) received either knowledge-graph-driven adaptive training or traditional uniform training covering the same syllabus. Training batches were matched on batch-level characteristics and allocated by random draw before enrollment. Because the two arms differ in delivery medium as well as in adaptive routing, the contrast estimates the effect of a bundled adaptive delivery system rather than adaptivity in isolation. Training self-efficacy was measured at T1 and T2, whereas transfer intention and knowledge were measured at T1, T2, and at the 6-week follow-up (T3). Cognitive load and perceived personalization fit were measured at T2, and measurement invariance across conditions was confirmed by confirmatory factor analysis. Linear mixed models showed small but robust advantages in training self-efficacy (*d* ≈ 0.26), transfer intention (*d* ≈ 0.25), and knowledge (*d* ≈ 0.29 at T2), with all effects surviving three clustering levels, a company-level wild cluster bootstrap, and a stratum-level permutation test. No reduction in the knowledge advantage was observed at follow-up, and equivalence testing bounded any change to less than 0.10 SD (T3 *d* ≈ 0.26). Parallel mediation showed statistical indirect effects of reduced extraneous load and higher perceived personalization fit on self-efficacy and knowledge, whereas the pathway to transfer intention ran through fit alone. Cognitive-load subscales diverged: adaptive training reduced extraneous load (*d* = −0.34) but increased intrinsic load (*d* = 0.28), partially supporting the load hypothesis and cautioning against treating cognitive load as a unitary construct. Exploratory moderation indicated that more experienced employees derived greater benefits, contrary to the compensatory pattern reported for novice workers. Findings extend cognitive load theory and personalization research to a high-reliability industrial workforce and suggest that the motivational returns of adaptive training are tied to employees' perceptions that the training fits them.

## 1 Introduction

Electric power utilities face a persistent training challenge. Grid technologies, safety regulations, and digital operating procedures change faster than traditional classroom-style training can be redesigned, while the workforce that must absorb these changes is large and heterogeneous in age, tenure, and prior digital experience. Uniform, one-size-fits-all delivery—still the default in most state-owned enterprises—treats a 25-year-old distribution engineer and a 50-year-old maintenance foreman as identical learners. AI-driven adaptive learning platforms that use knowledge graphs and learner models to route each employee through personalized content sequences promise a way out. Meta-analytic evidence from educational settings is encouraging: intelligent tutoring systems outperform conventional classroom instruction with effect sizes around *g* = 0.42–0.57 (Ma et al., 2014), and digital learning tools in secondary education average *g* = 0.65 (Hillmayr et al., 2020). Yet this evidence base has a conspicuous blind spot. It draws almost entirely on students—schoolchildren and undergraduates—in educational institutions. Whether adaptive training works for in-service employees embedded in real organizations, with real production constraints and real stakes, remains a question the literature has barely begun to address (Zhu et al., 2024; Brynjolfsson et al., 2023).

The gap is not merely a population issue. Three deeper problems limit what existing research can tell organizations considering adaptive training. First, the causal mechanism is asserted far more often than tested. Reviews of AI-driven personalization consistently invoke cognitive load theory (Sweller, 1988)—adaptive sequencing should reduce the load imposed by poorly matched content—but also note that formal statistical mediation tests of this pathway are rare (Gkintoni et al., 2025; Halkiopoulos and Gkintoni, 2024). Second, the candidate mechanisms have never been compared. Adaptive systems may work because they lighten cognitive processing (a cognitive pathway) or because employees perceive the training as tailored to them (a motivational pathway rooted in person—environment and task—technology fit logic) (Goodhue and Thompson, 1995; Walkington, 2013). They may operate through both pathways. No study we are aware of has tested these two pathways as parallel mediators within the same model, so their relative contributions remain unknown (Yüce et al., 2019). Third, outcome measurement in the adaptive-learning literature is dominated by immediate post-test scores. Organizational training research has long emphasized retention over time and the intention to transfer learning to the job (Baldwin and Ford, 1988; Blume et al., 2010), yet multi-wave follow-up designs are scarce, and transfer intention is essentially absent from adaptive-learning studies.

The present study addresses these gaps through a three-wave, matched-pair, cluster-randomized field experiment conducted within a Chinese provincial power utility, comparing knowledge-graph-driven adaptive training with traditional uniform training using an identical syllabus. Field settings of this kind satisfy the criteria that Harrison and List (2004) argue are required for externally valid causal inference—real participants, real tasks, real stakes—and remain exceptional in this literature: the closest antecedents are a quasi-experiment on adaptive microlearning with 111 in-service personnel (Zhu et al., 2024) and a cluster-randomized cognitive-ergonomics intervention in Finnish workplaces (Kalakoski et al., 2020), neither of which examined AI-driven adaptive training at scale in a high-reliability industrial workforce.

### 1.1 Theoretical background and hypotheses

#### 1.1.1 Main effects of adaptive training

Cognitive load theory holds that learning is constrained by limited working-memory capacity (Miller, 1956; Sweller, 1988): instructional designs that waste capacity on processing irrelevant to schema construction (extraneous load) impair learning, whereas designs aligned with the learner's current knowledge free capacity for generative processing (Chi et al., 1989). Adaptive platforms operationalize this alignment continuously—diagnosing knowledge state, selecting the next node in the knowledge graph, and adjusting difficulty. Consistent with meta-analytic evidence in education (Ma et al., 2014; Hillmayr et al., 2020) and the single workplace quasi-experiment available (Zhu et al., 2024), we predicted:

H1. Relative to traditional uniform training, adaptive training yields higher (a) training self-efficacy at T2, (b) knowledge at T2 *and* at follow-up T3 (i.e., a durable rather than merely immediate advantage), and (c) transfer intention at T2.

#### 1.1.2 Two parallel mediating pathways

Why should delivery format change these outcomes? The cognitive pathway follows directly from cognitive load theory: uniform delivery forces mismatched learners to process content that is either redundant or premature, inflating extraneous load; adaptive sequencing removes this mismatch. Reduced load should cascade into better knowledge acquisition, stronger mastery experiences—the primary source of self-efficacy (Bandura, 1977)—and greater willingness to apply what was learned.

H2. Adaptive training improves (a) training self-efficacy, (b) knowledge, and (c) transfer intention indirectly through lower cognitive load—specifically, we expected reductions in intrinsic and extraneous load, with no directional prediction for germane load. Because theory and evidence suggest the three load types need not move together—indeed the independence and measurement of germane load remain actively debated (Sweller, 2010; Kalyuga, 2011; Krieglstein et al., 2023; Zeitlhofer et al., 2024; Mo et al., 2022)—we specified extraneous load as the focal mediator and examined intrinsic and germane load separately.

The motivational pathway is distinct, and joins two research traditions. Person—environment fit research shows that the perceived match between a person and their work environment carries broad motivational consequences (Kristof-Brown et al., 2005); Walkington (2013) showed experimentally that content personalized to learners' interests improves performance; and task—technology fit research argues that perceived fit between a technology and one's task needs drives use and performance partly through motivational intermediaries (Goodhue and Thompson, 1995; Yüce et al., 2019). An employee who experiences training as “built for me”—right level, right pace, relevant to their job role—gains a sense of being recognized by the organization's systems, which should raise efficacy beliefs and transfer motivation independently of any change in processing load. Critically, personalization can fail to improve outcomes even when it changes learning processes (Lim et al., 2022), and most personalization research never measures the learner's perception of fit at all (Bartolomé et al., 2018), treating the manipulation as self-evidently experienced. We measured perceived personalization fit directly and predicted:

H3. Adaptive training improves (a) training self-efficacy, (b) knowledge, and (c) transfer intention indirectly through higher perceived personalization fit.
H4. The two pathways operate in parallel. As a non-directional, exploratory contrast, we tested whether their contributions differ in magnitude for each outcome (bootstrap confidence interval on the difference between the two indirect effects).

#### 1.1.3 Who benefits more?

Theory admits two opposing answers. Evidence from generative-AI assistance at work shows the largest gains for novice, low-skill workers (Brynjolfsson et al., 2023), and personalization effects in education are often strongest for struggling learners (Walkington, 2013): adaptive training may compensate for weaker prior foundations. The expertise reversal effect points the other way (Kalyuga, 2007): instruction tailored to the learner pays off for *experienced* learners precisely when it strips away redundant material that uniform formats force on them, so adaptivity could instead amplify the advantage of richer prior schemas. Following the workplace-AI evidence, we predicted compensation:

H5. The advantage of adaptive over uniform training is stronger for employees with shorter tenure (a compensatory pattern rather than a Matthew effect). This hypothesis was designated secondary/exploratory in the pre-specified analysis plan.

Figure 1 summarizes the hypothesized model.

**Figure 1:**
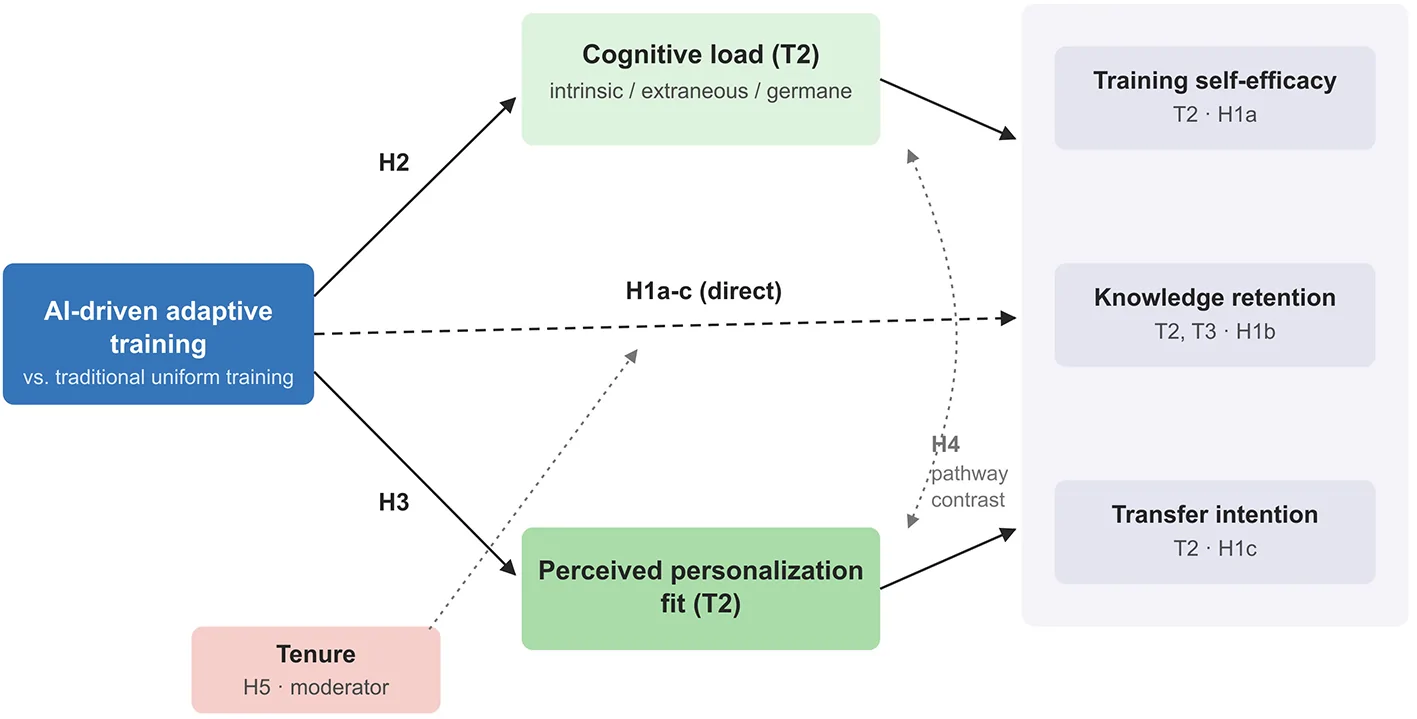
Hypothesized model. Adaptive vs. traditional training is expected to improve training self-efficacy (H1a), knowledge retention (H1b), and transfer intention (H1c), in parallel through cognitive load (H2) and perceived personalization fit (H3); H4 contrasts the two indirect pathways, and H5 posits tenure as a moderator of the training effect. Solid arrows denote hypothesized indirect pathways, the dashed arrow the direct effect, and dotted arrows the pathway contrast and moderation.

## 2 Materials and methods

### 2.1 Design and setting

We conducted a two-condition (adaptive vs. traditional training) matched-pair cluster-randomized field experiment with three measurement waves: T1 (before training), T2 (immediately after the training block), and T3 (follow-up approximately 6 weeks after training). The study was conducted as a research project of the School of Management at Zhengzhou University, in collaboration with the human resources department of State Grid Zhengzhou Power Supply Company, a provincial subsidiary of a large state-owned electric power corporation in central China. It was implemented in six of its county-level power supply companies. Within each company, 500 employees scheduled for the same annual technical-refresher curriculum were enrolled (250 per condition per company).

The study was embedded in the existing training program rather than mounted as a purpose-built experiment, which shaped what the design could and could not control. The utility's own objective was to improve its annual refresher training; the research team's role was to allocate conditions, fix the measurement schedule, and analyze outcomes within an arrangement the organization was going to run regardless. Dedicated research batches, purpose-built classes, and recruitment of volunteers into a separate experimental protocol were not available options: employees attend the annual curriculum as part of their duties, batches are formed by the training department around facility and shift constraints, and parallel structures created for research purposes would not have been permitted. The design is therefore pragmatic in the sense of Schwartz and Lellouch (2026)—conducted under routine operating conditions rather than under conditions arranged for inference—and several features described below follow directly from that: assignment had to be at the level of intact batches, batch sizes track room capacity rather than a design parameter, batch composition follows the training department's scheduling, and the administrative records generated along the way were kept to operational rather than research standards.

#### 2.1.1 Assignment mechanism

The unit of assignment was the training batch, the intact group that attends the same sessions at the same site. Individual randomization was organizationally infeasible, and batch-level assignment also limited contamination between conditions. Before any T1 measurement, the HR department supplied the complete roster of scheduled batches, including batch size, job family, mean tenure, and prior-training records. Batches were then matched into pairs on these batch-level characteristics within company and job family, and within each matched pair the research team drew random numbers to assign one batch to the adaptive and the other to the traditional condition. Allocation was performed by the research team rather than by line managers, the training vendor, or the platform provider; it was fixed and recorded in a written allocation ledger before enrollment and before any outcome was measured; and no batch-level characteristic—performance history, supervisor preference, site, or schedule convenience—was permitted to determine which arm a batch entered. The allocation rule and ledger template are reproduced in Supplementary material S1.

#### 2.1.2 Design terminology

Because conditions were assigned by the research team via a random draw within matched pairs of batches, fixed in a written ledger before enrollment and before any outcome was measured, the design is a matched-pair cluster-randomized field experiment, and we label it as such throughout. Earlier versions of this report used the more conservative label “field quasi-experiment” for a different reason: the randomization cannot be verified in the analysis dataset because batch identifiers were not carried into the anonymized export. As a result, neither the assignment clusters nor the matched-pair structure is observable to the analyst, even though both governed allocation. That is a constraint on what the analysis can condition on, not on how conditions were allocated, and the two are kept separate below–the assignment mechanism is randomized at the batch level, whereas inference must proceed under assumptions about clusters we cannot observe. Training-batch records at each site are archived to operational rather than research standards: batch sizes tracked room capacity, which varied from roughly a dozen seats to about 100 across sites, and batches were merged and split during delivery as scheduling required. No exact batch-level count has been recovered from those records, and the batch totals reported below are accordingly given as an approximation rather than as a documented count. Accordingly, the randomization is not documentarily verifiable from the analysis dataset beyond the researchers' contemporaneous allocation ledger; we retain the matched-pair cluster-randomized label based on that ledger and the procedure it documents—random draw by the research team, within matched pairs, fixed before enrollment—not on independent third-party corroboration. We flag this as a documentation limit rather than a design one. We also correct an earlier characterization for the record: a previous version of this manuscript's Limitations section described the assignment mechanism itself as “cluster-based and non-random.” That description was inaccurate and is withdrawn here. Assignment was randomized—by the procedure just described—and the correct limitation, stated in the Limitations section below, is that this randomization cannot be independently audited from the anonymized analysis dataset, not that it did not occur.

#### 2.1.3 Batch structure and its treatment in analysis

Batch size was not set by the research design but by the seating capacity of the training facility at each site, which ranged from roughly a dozen seats at the smallest county locations to about 100 at the largest, so batches were markedly unequal in size; this capacity range describes the variability in batch size, not the total batch count, which we report separately below. The total batch count is an approximation, not a figure retrieved from the archived records. Scheduling at each site ran to about five to eight batches per condition per company, which puts the total at roughly 65–100 across the six companies and two conditions among the 3,000 enrolled employees. We label this an approximation deliberately: the training department's records are archived to operational standards and do not yield an exact batch-level count, so the figure reflects the scale at which scheduling operated rather than a count read off a ledger. It is not derived from the facility-capacity range just given, which describes how batch *size* varied rather than how many batches there were. Allocation was carried out on the batches as they stood in the ledger at that point. During implementation, however, room capacity and schedule changes led to batches being merged and split *within* condition, so the groups that actually attended together no longer map one-to-one onto the batches recorded at allocation; merging across conditions was not possible, since the two arms were delivered in different formats—platform-based individual sessions vs. classroom sessions—and never shared a session. Raw batch identifiers were therefore not carried into the anonymized data export. After these operational changes, they no longer identified the units on which allocation had been performed, and the exact batch membership of individual employees cannot be reconstructed. Attendance registers and facility scheduling records do exist at each site, so a session-by-session reconstruction is not in principle impossible; it would, however, require manual collation of archived administrative records for 3,000 employees across six separate companies, and would recover the groups that attended together rather than the units on which allocation was performed. The design fixes the allocation mechanism, which operated on batches; the data cannot deliver a clean *post-hoc* mapping from employees back to those batches. Because assignment happened at a level the analysis cannot observe, inference here does not rest on a single clustering assumption. First, models use the organizational strata within which batches were formed—company × job-family units (30 units, e.g., the operations-and-maintenance staff of one county company; 5 job families per company; unit sizes 39–152)—as random intercepts or as fixed effects with cluster-robust standard errors, and mediation inference uses cluster bootstrap over the 60 company × job-family × condition cells (cell sizes 16–76). Second, we re-estimate every primary effect with standard errors clustered at three successively coarser levels (60 cells, 30 units, 6 companies), the coarsest of which aggregates each county company into a single cluster, using Webb six-point wild cluster bootstrap-*t* at that level to accommodate the small number of company clusters (Cameron et al., 2008; Roodman et al., 2019). Coarser clustering does not mechanically inflate standard errors, so we report these as sensitivity analyses under alternative assumptions about the unobserved clusters rather than as a conservatism ordering. Third, we conduct a stratum-level permutation test in which condition labels are re-drawn within each company × job-family unit. Because the matched-pair structure is not recoverable, this permutation scheme approximates, rather than exactly reproduces, the matched-pair randomization actually used. We report it as a sensitivity analysis rather than as design-based randomization inference. All three are reported in Section 3.8.2.

### 2.2 Training conditions and implementation fidelity

Both conditions covered the same syllabus (grid operation and maintenance procedures, safety regulation updates, and digital operation tools) over the same 2-week calendar window at each company, with the same total scheduled hours, the same terminal assessment standard, and the same pool of instructors and materials writers. This alignment was verified before implementation through a signed content-alignment checklist. The traditional condition received the syllabus as uniform classroom sessions plus fixed-sequence e-learning modules, in which all learners moved through the same content in the same order at the same pace. The adaptive condition received the same content through the company's knowledge-graph-driven learning platform.

Technically, the platform represents the curriculum as a directed knowledge graph, with nodes as curriculum knowledge points and edges encoding prerequisite relations specified by the curriculum experts who wrote the syllabus. An entry diagnostic assessment initializes a mastery estimate for each node. The platform then selects the next node from the set whose prerequisites are estimated as mastered, calibrates item difficulty to the current mastery estimate, updates that estimate after each response, and reroutes the learner back to prerequisite nodes when performance on a node falls below the threshold. Telemetry exported for the adaptive arm records how much adaptation occurred: employees completed an average of 6.99 adaptive sessions (*SD* = 2.80, range 1–17) and experienced 3.62 path reroutings (*SD* = 1.89, range 0–10), with 97.1% of employees experiencing at least one rerouting.

Because the two arms differ in delivery medium and adaptivity, the contrast estimated here is that of a *bundled adaptive delivery system* vs. standard uniform training, not an isolated effect of the adaptive algorithm. Table 1 makes the bundle explicit by separating the dimensions that were held equivalent by design from those that necessarily co-vary with adaptive delivery, and it reports the implementation indicators available in the anonymized export. “Adaptive training” is used below as shorthand for this bundle.

**Table 1:** Intervention comparability and implementation fidelity.

Dimension	Adaptive condition	Traditional condition
Held equivalent by design (verified by signed content-alignment checklist)		
Syllabus and knowledge modules	Identical curriculum	Identical curriculum
Calendar window	Same 2-week block per company	Same 2-week block per company
Total scheduled hours	Matched to traditional arm	Matched to adaptive arm
Terminal assessment standard	Same	Same
Instructors/materials writers	Same pool	Same pool
Technical support channel	In-platform Q&A plus named contact	Instructor on site
Training completion	99.5%	99.3%
Measurement burden (median questionnaire duration, T1/T2/T3)	1,242 / 1,762 / 901 s	1,248 / 1,767 / 900 s
Co-varies with adaptive delivery (the bundle)		
Delivery medium	Platform-based, individually paced	Instructor-led classroom plus fixed e-learning
Instructor contact	Reduced; asynchronous support	Continuous during classroom sessions
Entry diagnostic	Yes (initializes mastery model)	No
Content sequencing	Individually routed over knowledge graph	Fixed for all learners
Item difficulty	Calibrated to current mastery estimate	Uniform
Embedded retrieval practice/feedback	Continuous, item-level, immediate	Periodic, at module level
Learner pacing	Self-paced within the block	Instructor-paced
Platform exposure	6.99 sessions (*SD* = 2.80); 3.62 re-routings (*SD* = 1.89); 97.1% re-routed at least once	Fixed-sequence e-learning only; no adaptive routing

Dimensions above the rule were held equivalent by design; dimensions below it co-vary with adaptive delivery and are part of the bundle under test.

### 2.3 Participants

Of 3,000 enrolled employees, 2,577 (85.9%) consented and completed T1. Following the pre-specified exclusion rules (attention-check failure or straight-lining *combined with* anomalously fast completion), 3 respondents were excluded, leaving an analysis sample of *N* = 2, 574 (1,284 adaptive, 1,290 traditional). In the adaptive arm, 1,243 (96.8% of the analysis sample) provided T2 data and 1,200 (93.5%) provided T3 data, the latter representing a further 43 employees lost after T2; the corresponding figures in the traditional arm were 1,251 (97.0%) at T2, 1,206 (93.5%) at T3, and 45 lost after T2. T3 measurement was open to the entire analysis sample regardless of T2 completion, but in practice no employee who missed T2 returned at T3, so 2,406 employees (93.5%) provided data at all three waves. Mean age was approximately 38 years, mean tenure approximately 12 years; the sample spanned engineering, operations and maintenance, marketing, and support job families. Figure 2 shows the participant flow. Participation in the research measurements was voluntary and independent of the training itself, under the safeguards detailed in the Ethics Statement.

**Figure 2:**
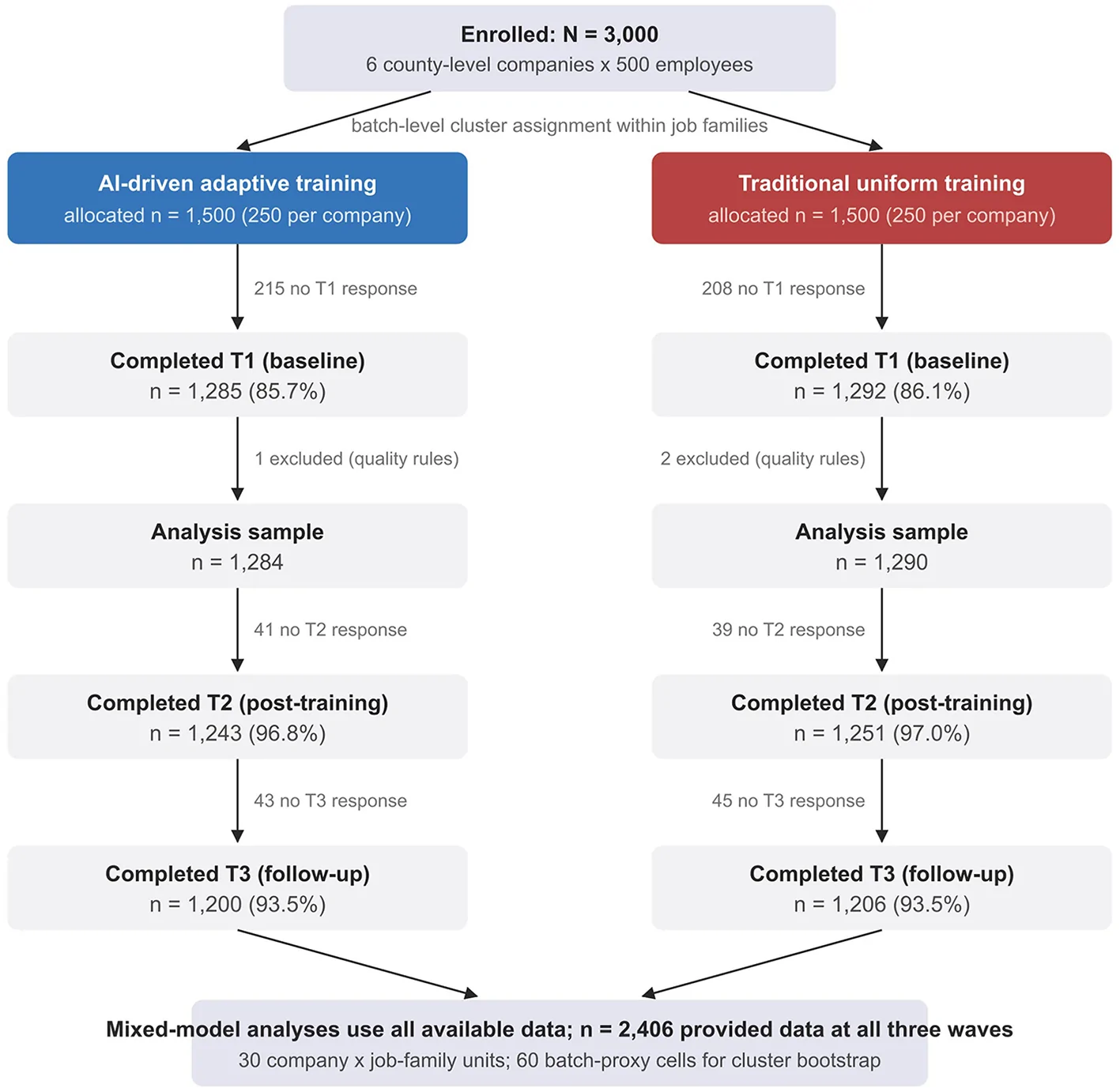
Participant flow through the study. Within each of the six county-level companies, 250 employees per condition were allocated at the training-batch level within job families. Retention from the analysis sample was 96.8%/97.0% at T2 and 93.5%/93.5% at T3 in the adaptive/traditional conditions.

### 2.4 Measures

Unless noted, self-report items used 7-point Likert scales (1 = *strongly disagree*, 7 = *strongly agree*) and were administered in Chinese. Composites are item means.

Cognitive load (T2): Ten items adapted from the three-factor instrument of Leppink et al. (2013), using the validated Chinese version (Zhang et al., 2020), rated 0–10: intrinsic load (3 items, α = 0.68), extraneous load (3 items, α = 0.68), and germane load (4 items, α = 0.73). The three-factor structure follows Krieglstein et al. (2023) in treating load types as conceptually distinct.

Perceived personalization fit (T2): Six purpose-built items were developed for this study because no published instrument measures the learner's *perception* of personalization: personalization research typically manipulates fit without measuring whether learners experience it (Walkington, 2013; Bartolomé et al., 2018). Development proceeded from a construct definition—an employee's subjective judgment that the training's content difficulty, job relevance, learning path, pace, and responsiveness match their own level, post requirements, and learning needs—to a content-domain map with six facets (level match, job-requirement match, recognition of individual weak points, overall customization, path and pace match, and responsive support), with one item per facet. Items were worded for the electric-power training context and reviewed by the curriculum experts who wrote the syllabus. Items are answered on the same 7-point scale by both conditions and make no reference to any platform, so that traditional-arm employees can report low or high fit on identical terms (e.g., “This training felt tailor-made for me rather than one-size-fits-all”). All six items in English and Chinese, with facet mapping and scoring rules, appear in Supplementary material S2; α = 0.83.

Training self-efficacy (T1, T2): Seven items (one reverse-scored) adapted from the general self-efficacy template (Zhang and Schwarzer, 1995; Bandura, 1977) to the training domain, with items written to span Bandura's efficacy sources (mastery experience, generalization to future learning, autonomous problem solving); α = 0.82 at both waves. Full items are in Supplementary material S2.

Transfer intention (T1, T2, T3): Six items based on the theory of planned behavior (Ajzen, 1991) and the training-transfer tradition (Baldwin and Ford, 1988), measuring behavioral intention, attitude toward applying the content, intention strength, and overall transfer intention; α = 0.82–0.83. Full items are in Supplementary material S2.

Measurement model and invariance: Two of the focal scales are purpose-built, and the fit—transfer dissociation reported below depends on the constructs being empirically separable. We fitted a confirmatory factor analysis to the full 29-item T2 self-report battery (three cognitive-load factors, fit, self-efficacy, transfer intention; MLR estimation, *n* = 2, 318 complete responses). The six-factor model fitted well, χ^2^(362) = 411.3, CFI = 0.997, TLI = 0.997, RMSEA = 0.008, SRMR = 0.018, with all standardized loadings significant (fit 0.54–0.77; self-efficacy 0.24–0.78; transfer intention 0.43–0.79; load subscales 0.49–0.78). The single weak loading, 0.24, belongs to the one reverse-keyed self-efficacy item (TSE7, “Faced with the content of this training, I often worry that I will not be able to learn it”), which is the familiar method effect of reverse keying rather than substantive misfit; its corrected item—total correlation with the remaining six items is −.22. Dropping it improves the measurement model on every index—the six-factor solution then fits χ^2^(335) = 343.0, CFI = 1.000, RMSEA = 0.003, SRMR = 0.016, self-efficacy loadings run 0.60–0.78, composite reliability rises from 0.83 to 0.85, average variance extracted from 0.42 to 0.48, and α from 0.82 to 0.84, with configural, metric, and scalar invariance across conditions still supported—and it leaves the substantive results unchanged: the six-item composite correlates 0.98 with the seven-item composite, and the condition effect on T2 self-efficacy is *b* = 0.31 (SE = 0.04, *p* < 0.001, *d* = 0.25) against *b* = 0.30 (*d* = 0.26) with all seven items, with both indirect effects, the pathway contrast, and the condition × tenure interaction (*b* = 0.032 per year, *p* < 0.001) likewise unchanged. The seven-item version is retained because it is the pre-specified scoring; the full item-by-item comparison is in Supplementary material S3.6. Composite reliabilities were 0.84 (fit), 0.83 (self-efficacy), 0.84 (transfer intention), and 0.69–0.73 for the load subscales. Average variance extracted ranged from 0.41 to 0.47 and thus fell below the conventional 0.50 benchmark. With composite reliabilities above 0.70, this is usually judged acceptable convergent validity (Fornell and Larcker, 1981). Discriminant validity was unambiguous by the Fornell–Larcker criterion, since the largest factor correlation in the model (*r* = 0.42, self-efficacy with transfer intention) is well below the smallest square root of AVE (0.64). Two competing specifications were also tested. Merging perceived fit and transfer intention into a single factor severely degraded fit (χ^2^(367) = 3, 956.2, CFI = 0.810, RMSEA = 0.065, ΔCFI = −0.187), and a single-factor model of the entire battery fitted very poorly (CFI = 0.437, RMSEA = 0.111). The first comparison argues against semantic redundancy between fit and transfer intention. The second establishes that the six constructs are not reducible to one factor, which is necessary but not sufficient to rule out common-method bias: a poorly fitting one-factor solution constrains, rather than eliminates, the possibility of shared method variance among concurrently administered self-reports, and we treat method variance as bounded rather than excluded. Heterotrait–monotrait ratios show the same pattern (Henseler et al., 2015): fit with transfer intention 0.35, with training self-efficacy 0.33, and with extraneous load 0.02, all far below the 0.85 threshold.

Multi-group CFA tested whether the battery measures the same constructs in the same metric in both conditions, a precondition for the between-condition comparisons and *b* paths reported below. Configural (CFI = 0.995, RMSEA = 0.010, SRMR = 0.024), metric (CFI = 0.996, ΔCFI = 0.000), and scalar models (CFI = 0.996, ΔCFI = 0.000, ΔRMSEA = 0.000) were all well fitting, and the metric and scalar constraints cost essentially nothing, within the |ΔCFI| < 0.010 convention (Cheung and Rensvold, 2002). Full loading, reliability, and invariance tables are in Supplementary material S3.

Knowledge (T1, T2, T3): A 20-item objective test built by curriculum experts to cover the syllabus knowledge modules, automatically scored (0–20). Parallel forms constructed from a common blueprint—same module weights, same item formats, same target difficulty band—were used across waves to limit testing effects. Form-level statistics support equivalence: mean item difficulty was 0.51 (*SD* = 0.18, range 0.24–0.79) at T1, 0.53 (*SD* = 0.18, range 0.26–0.84) at T2, and 0.55 (*SD* = 0.18, range 0.26–0.84) at T3, with no detectable difference in difficulty distributions between the T1 and T2 forms, *t*(38) = −0.44, *p* = 0.66, or between the T2 and T3 forms, *t*(38) = −0.22, *p* = 0.83. Mean point-biserial discrimination was stable (0.35, 0.34, 0.35) as was internal consistency (KR-20 = 0.79, 0.78, .79). The later forms were, if anything, marginally easier than the earlier ones, and both conditions took the same form at each wave, so residual form differences do not enter the between-condition contrasts.

Covariates: Tenure (years), education, digital-device proficiency (3 items, self-report), prior training participation count, and the corresponding T1 baseline of each outcome. T2 questionnaires embedded an instructed-response attention check and a contamination probe (whether the respondent had discussed training content with members of the other condition; 1.4% affirmative).

### 2.5 Analytic strategy

The analysis plan was specified and locked internally before data collection. All tests of H1–H5 were specified in that plan; analyses added after seeing the data are labeled *post-hoc* at first mention and are listed as such in the deviation log (Supplementary material S4), which records every departure from the locked plan and every analysis added in response to peer review.

#### 2.5.1 Primary models

H1 was tested with linear mixed models. For the T2 single-time-point outcomes the model was $Y_{i}=\beta_{0}+\beta_{1}{\text{Condition}}_{i}+\beta_{2}Y_{i,T1}+\gamma^{\prime }{\text{X}}_{i}+u_{j\left[i\right]}+\varepsilon_{i}$, where **X** contains digital proficiency, tenure, education, and prior training count, and *u*_*j*_ is a random intercept for the company × job-family unit. For knowledge retention we fitted a repeated-measures model over T2 and T3, $Y_{it}=\beta_{0}+\beta_{1}{\text{Condition}}_{i}+\beta_{2}{\text{Time}}_{t}+\beta_{3}\left({\text{Condition}}_{i}\times {\text{Time}}_{t}\right)+\beta_{4}Y_{i,T1}+\gamma^{\prime }{\text{X}}_{i}+\delta^{\prime }{\text{U}}_{i}+v_{i}+\varepsilon_{it}$, with an individual random intercept *v*_*i*_ and unit fixed effects **U**, followed by the T3 simple effect. We controlled the false discovery rate across the H1–H3 primary tests with the Benjamini–Hochberg procedure (Benjamini and Hochberg, 1995), which bounds the expected proportion of false discoveries rather than the familywise error rate. H2–H4 used parallel multiple mediation (Preacher and Hayes, 2008) entering both mediators simultaneously, with percentile confidence intervals from 5,000 cluster-bootstrap resamples drawn over the 60 company × job-family × condition cells; the H4 path contrast is the bootstrap distribution of the difference between the two indirect effects. H5, designated secondary and exploratory in the locked plan, added condition × tenure interactions to the H1 models, with simple slopes evaluated at ±1 SD. Standardized effect sizes *d* are computed as the adjusted condition coefficient divided by the observed outcome standard deviation, and confidence-interval endpoints are transformed identically. Complete model specifications, including all covariates and random-effect structures, are provided in Supplementary material S4.

#### 2.5.2 Durability (*post-hoc*)

The × time interaction was pre-specified, but a nonsignificant interaction does not constitute evidence of no decay. Durability is therefore evaluated using two one-sided tests (Lakens et al., 2018) and a complementary non-inferiority test. Both were added in response to peer review; neither the smallest effect size of interest nor the non-inferiority margin appears in the locked analysis plan, so both are labeled *post-hoc* here and in the deviation log (Supplementary material S4). The smallest effect size of interest was set at 0.10 SD of the knowledge score, which on this instrument is 0.42 points—less than the value of one test item—on the substantive ground that a change in retained knowledge smaller than a single item of a 20-item test cannot be interpreted as decay for any training-evaluation decision this study is meant to inform. The non-inferiority margin, that the T3 effect retains at least half of the T2 effect, is the conventional halving point separating substantial retention from marked erosion; it is defined as a proportion of the estimated T2 effect and was fixed before the T3 estimate was inspected. Claims about persistence rest on these two tests; the interaction *p* value is reported descriptively.

#### 2.5.3 Inference under uncertainty about the assignment clusters

Since batch identifiers are unavailable, primary standard errors were complemented by three additional procedures at successively coarser levels of aggregation, all added *post-hoc* in response to review: cluster-robust standard errors at the 60-cell, 30-unit, and 6-company levels; Webb six-point wild cluster bootstrap-*t* at the company level, which is valid with few clusters (Cameron et al., 2008; Roodman et al., 2019); and a stratum-level permutation test in which condition labels are re-drawn within each company × job-family unit (2,000 permutations), which approximates but does not exactly reproduce the matched-pair scheme by which batches were actually allocated. We also retained the design-effect corrections for hypothetical batch-level intraclass correlations reported below.

#### 2.5.4 Robustness and sensitivity

Robustness checks included excluding attention-check failures and contamination-flagged cases, comparing fixed- and random-effects specifications with cluster-robust standard errors, conducting completer-only analyses, and respecifying the mediation model using composite loadings. Four additional sensitivity analyses addressed identification and measurement concerns: (a) longitudinal mediation models linking T2 mediators to T3 outcomes, which separate mediator and outcome in time, and an autoregressive variant that additionally controls for the T2 outcome; (b) multiple imputation (*m* = 20, iterative chained equations with posterior draws, Rubin's rules) compared with the available-case estimates; (c) false-discovery-rate correction within the family of six indirect effects (three outcomes × two mediators) using bootstrap *p* values; and (d) an errors-in-variables respecification that corrects the mediator *b* paths for unreliability, guarding against the possibility that the less reliable load measure loses the pathway comparison by attenuation alone. Analyses used Python with statsmodels 0.14 (Python Software Foundation, Wilmington, DE, United States) for regression, with confirmatory factor analyses in R with lavaan 0.6 (R Foundation for Statistical Computing, Vienna, Austria). The locked analysis plan, deviation log, full analysis outputs, and all analysis code are provided as Supplementary materials S4, S5; the de-identified analysis dataset is available from the authors under the data-sharing terms agreed with the host organization.

## 3 Results

### 3.1 Baseline equivalence, attrition, and descriptives

The conditions were closely balanced at baseline: standardized differences in age, tenure, education, digital proficiency, prior training count, gender, and all three T1 outcome baselines were |*d*| ≤ 0.08. The largest, tenure (*d* = 0.08, *p* = 0.047; adaptive *M* = 12.5 vs. traditional *M* = 12.0 years), reached nominal significance given the large sample; tenure was controlled in all models and is itself the H5 moderator, and we probe this imbalance further in the robustness checks. Attrition was low and symmetric (6.5% in both conditions by T3), and T3 dropouts did not differ from completers on any observed baseline variable (all *p*>0.14; joint likelihood-ratio test χ^2^(9) = 4.58, *p* = 0.87). These tests cannot establish that data are missing completely at random–unobserved causes of dropout are not ruled out by nonsignificant predictors–but they do show the absence of *differential* attrition on measured characteristics. Missingness that depends on observed data is handled by the multiple-imputation analysis reported below. Table 2 reports condition-level descriptive statistics for all focal variables.

**Table 2:** Descriptive statistics by condition and unadjusted standardized differences.

Variable	Adaptive *M* (SD)	Traditional *M* (SD)	*d*
Training self-efficacy T1	4.19 (1.15)	4.22 (1.16)	−0.03
Transfer intention T1	4.38 (1.25)	4.40 (1.16)	−0.02
Knowledge T1 (0–20)	10.14 (4.19)	10.21 (4.15)	−0.02
Training self-efficacy T2	4.42 (1.14)	4.15 (1.13)	0.24
Transfer intention T2	4.60 (1.21)	4.32 (1.22)	0.23
Knowledge T2	11.27 (4.03)	10.10 (4.09)	0.29
Transfer intention T3	4.59 (1.20)	4.33 (1.20)	0.21
Knowledge T3	11.46 (4.10)	10.40 (4.17)	0.26
Intrinsic load T2 (0–10)	5.73 (2.00)	5.17 (1.98)	0.28
Extraneous load T2	5.15 (2.00)	5.83 (2.02)	−0.34
Germane load T2	5.44 (1.92)	5.62 (1.91)	−0.10
Perceived personalization fit T2	4.32 (1.26)	4.15 (1.20)	0.14

### 3.2 Main effects (H1)

All three primary effects were supported and survived FDR correction. Adjusting for baselines and covariates with unit random intercepts, adaptive training increased T2 training self-efficacy [*b* = 0.30, SE = 0.04, 95% CI [0.22, 0.37], *p* < 0.001, *d* = 0.26, CI [0.20, 0.32]; H1a] and T2 transfer intention [*b* = 0.30, SE = 0.04, CI [0.22, 0.39], *p* < 0.001, *d* = 0.25, CI [0.18, 0.32]; H1c]. For knowledge (H1b), the repeated-measures model showed a condition effect of *b* = 1.15 points (SE = 0.15, *p* < 0.001) at T2, and the T3 simple effect remained *b* = 1.07 [SE = 0.15, *p* < 0.001, *d* = 0.26, CI [0.18, 0.33]; T2 *d* = 0.29, CI [0.22, 0.35]]. Figure 3 displays the knowledge trajectories.

**Figure 3:**
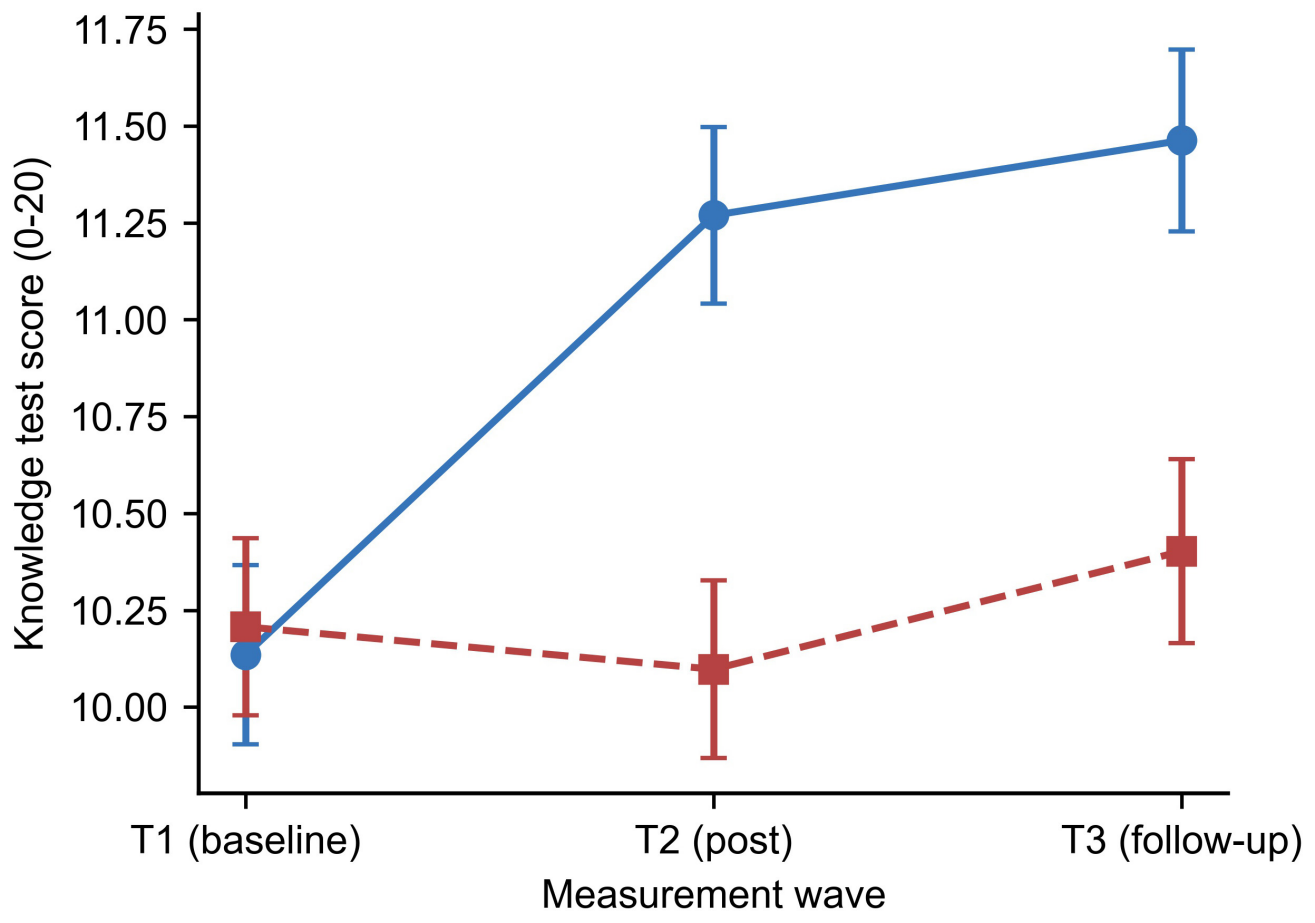
Knowledge test scores (0–20) across waves by condition (solid blue, adaptive training; dashed red, traditional training). Error bars are 95% confidence intervals of the mean. The groups are indistinguishable at baseline; the post-training advantage of the adaptive condition is still present at follow-up, with no statistically detectable change between T2 and T3 (equivalence supported within ±0.10 SD).

We assessed durability with equivalence testing rather than with the non-significant interaction alone. The condition × time interaction was *b* = −0.10 points [SE = 0.16, *p* = 0.52; 90% CI [−0.36, 0.16], i.e., [−0.09, 0.04] in SD units], and two one-sided tests against a smallest effect size of interest of 0.10 SD (0.42 points) supported statistical equivalence, *p* = 0.021; equivalence also held against wider bounds of 0.15 SD (*p* < 0.001). The complementary non-inferiority test showed that the follow-up advantage retained at least half of the immediate advantage, *z* = 3.05, *p* = 0.001. Over this 6-week window, then, no reduction in the adaptive advantage was detected, and any true reduction is bounded below 0.10 SD.

### 3.3 Within-condition change and the absence of a gain under uniform training

Within-person change across waves is as follows. In the adaptive arm, knowledge rose by 1.14 points from T1 to T2 [95% CI [0.91, 1.37], *t*(1, 182) = 9.66, *p* < 0.001, *d*_*z*_ = 0.28] and by 1.33 points from T1 to T3 (*p* < 0.001). In the traditional arm, by contrast, no gain was detectable at T2 [−0.07 points, CI [−0.29, 0.16], *t*(1, 198) = −0.59, *p* = 0.56] and only a small, nonsignificant gain by T3 (+0.20 points, *p* = 0.11).

The form statistics reported in the Measures section directly inform the interpretation of this null. Parallel-form drift is ruled out: the T2 form was, if anything, marginally easier than the T1 form (mean item difficulty 0.53 vs. 0.51; difficulty distributions were statistically indistinguishable, *p* = 0.66), so a harder post-training form is not masking a traditional-arm gain. Discrimination and internal consistency were stable across forms. Insufficient test sensitivity is also unlikely, since the same instrument detected a reliable gain of comparable size in the adaptive arm and discriminated well at every wave. The substantive reading remains: a 2-week uniform refresher on material that experienced employees have encountered before produced no measurable knowledge gain in this workforce, consistent with long-standing evidence that annual refresher training in high-reliability industries often fails to move objective knowledge (Baldwin and Ford, 1988; Blume et al., 2010). The adaptive contrast here is thus a contrast between a format that produced learning and one that, on this syllabus and in this workforce, did not. Both arms took the same form at each wave, so this asymmetry does not enter any between-condition estimate.

### 3.4 Cognitive load pattern

The load subscales moved in different directions (Figure 4). Adaptive training reduced extraneous load (*d* = −0.34), as theorized, but *increased* intrinsic load (*d* = 0.28) and slightly reduced germane load (*d* = −0.10). Consequently, the composite load score showed almost no group difference (*d* = −0.09), illustrating how aggregation can mask offsetting subscale effects. H2 predicted reductions in both intrinsic and extraneous load, so this pattern supports the hypothesis only in part: the focal extraneous-load prediction held, while the intrinsic-load prediction was reversed. H2 is therefore partially supported, and the intrinsic-load reversal is discussed in the Discussion.

**Figure 4:**
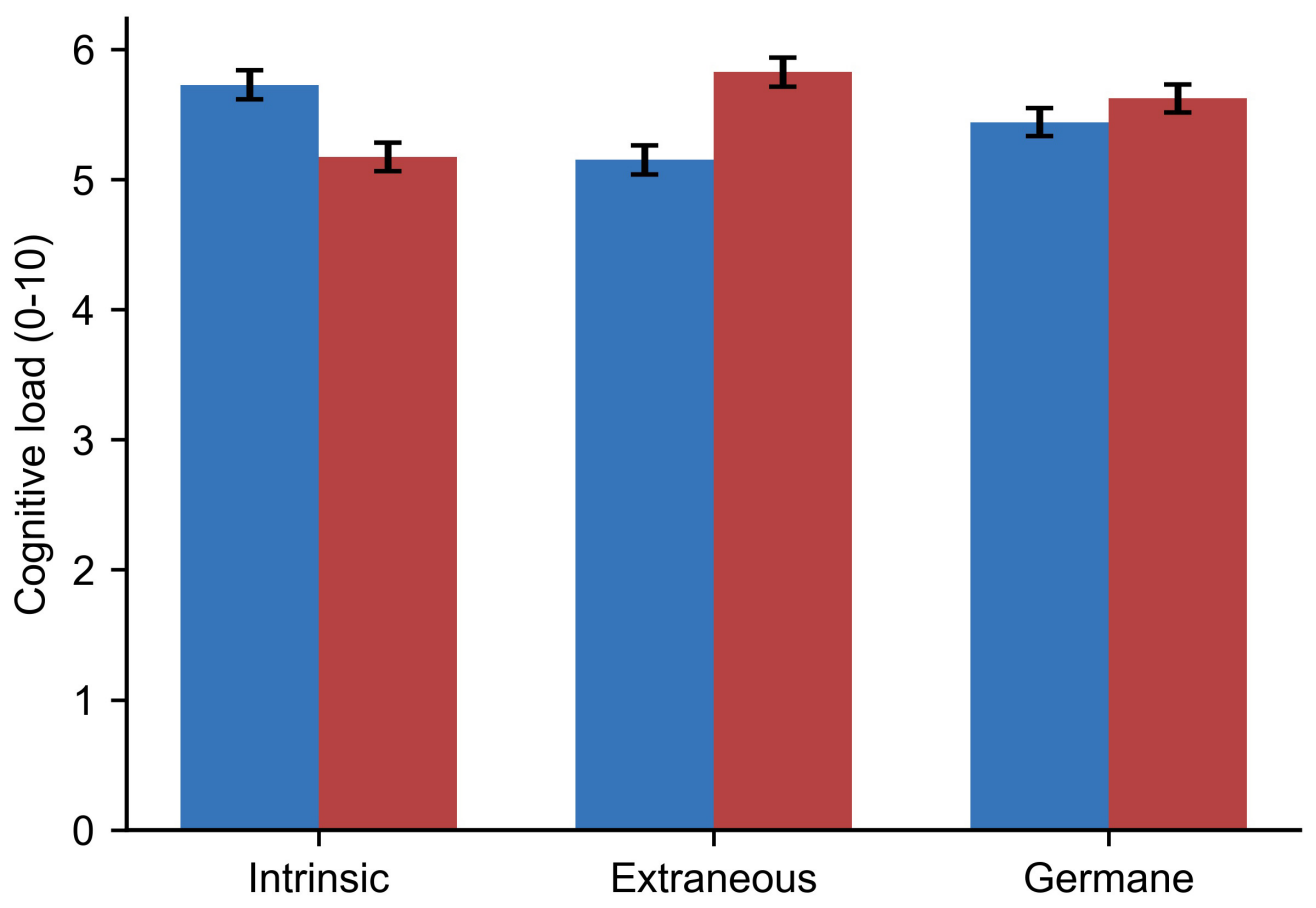
Cognitive load subscale means (0–10) by condition (blue, adaptive training; red, traditional training). Error bars are 95% confidence intervals. Adaptive training reduces extraneous load but raises intrinsic load, with little difference in germane load.

### 3.5 Parallel mediation (H2–H4)

Table 3 summarizes the parallel mediation models, and Figure 5 displays the estimated paths. These models estimate statistical indirect effects: the condition contrast is experimental at the batch level, but the mediators were measured rather than manipulated, so the *b* paths are associations among measured variables and the products *a*×*b* are not identified causal mechanisms. Adaptive training reliably reduced extraneous load (*a*_1_ ≈ −0.69) and increased perceived personalization fit (*a*_2_ ≈ 0.19) in all models. For training self-efficacy, both indirect effects were significant [via extraneous load: 0.026, CI [0.012, 0.043]; via fit: 0.029, CI [0.014, 0.045]], with no difference between pathways. For knowledge, both were again significant [via load: 0.092, CI [0.036, 0.163]; via fit: 0.050, CI [0.019, 0.089]], with no significant pathway difference. For transfer intention; however, the load pathway was null—extraneous load did not predict transfer intention controlling for fit (*b*_1_ = −0.004, *p* = 0.59)—while the fit pathway remained significant [0.041, CI [0.019, 0.065]], and the pathway contrast was significant [−0.039, CI [−0.067, −0.011]]. Thus, the load-mediated predictions H2a and H2b were supported and H2c was not—which, together with the reversed intrinsic-load effect reported above, makes H2 partially supported overall—while H3a–c were supported, and the pathway asymmetry probed by the exploratory H4 contrast emerged specifically for the motivational outcome. Direct effects remained significant throughout, indicating that the two measured pathways together account for only part of the condition effect.

**Table 3:** Parallel mediation of condition effects through extraneous cognitive load (CL-E) and perceived personalization fit (PFP): unstandardized statistical indirect effects with 5,000-resample cluster-bootstrap percentile CIs.

Outcome	Via CL-E [95% CI]	Via PFP [95% CI]	Difference [95% CI]	Direct *c*^′^
Self-efficacy T2	0.026[0.012, 0.043]	0.029[0.014, 0.045]	−0.003[−0.023, 0.018]	0.238
Knowledge T2	0.092[0.036, 0.163]	0.050[0.019, 0.089]	0.042[−0.022, 0.115]	1.031
Transfer intention T2	0.003[−0.012, 0.018]	0.041[0.019, 0.065]	−0.039[−0.067, −0.011]	0.256

**Figure 5:**
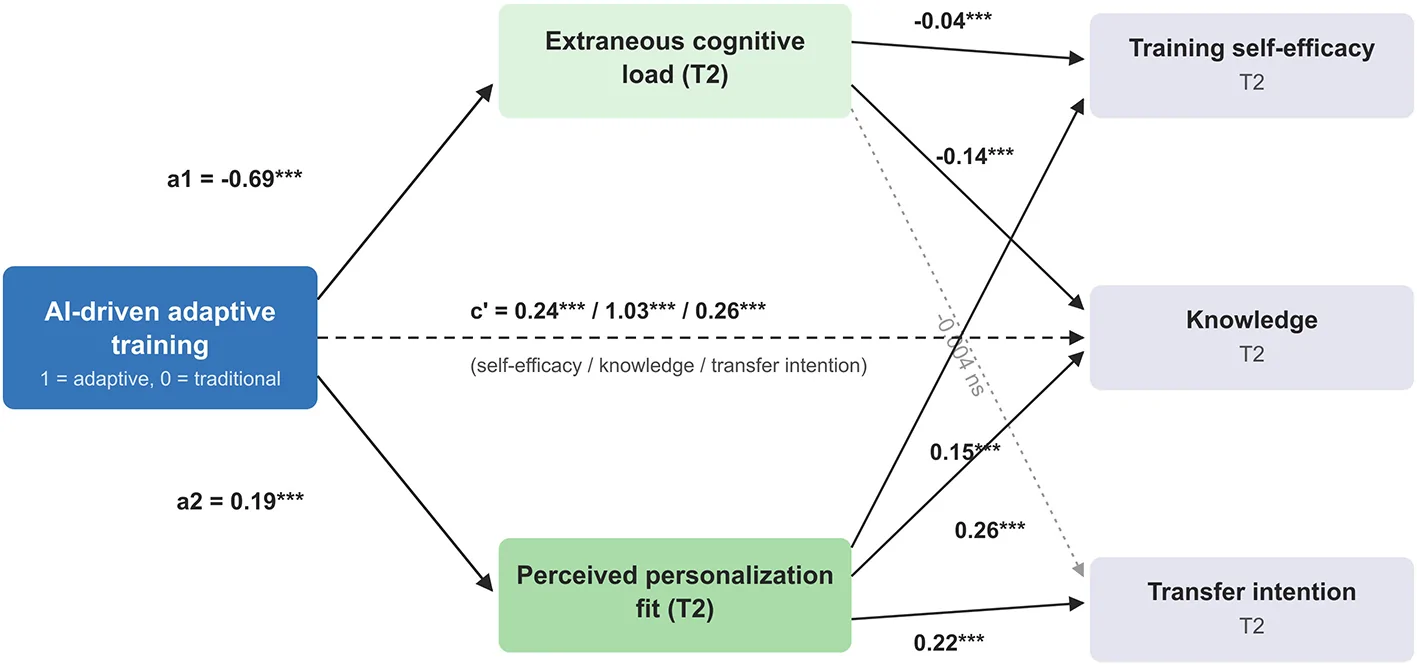
Estimated parallel mediation model. Unstandardized coefficients from the models in Table 3; *b* paths and direct effects *c*′ are reported per outcome in the order self-efficacy/knowledge/transfer intention, where a single arrow serves all three outcomes. The gray dotted path (extraneous load to transfer intention) is nonsignificant; all other paths *p* < 0.001. ^***^p < 0.001; ns, non-significant.

Pre-specified exploratory decomposition across all three load types showed that the extraneous pathway was the only load pathway operating in the theorized direction; the elevated intrinsic load in the adaptive condition carried small *negative* indirect effects on self-efficacy and knowledge (e.g., *a*×*b* = −0.044 for knowledge), partially offsetting the extraneous-load benefit—a suppression pattern invisible to composite-load analyses.

### 3.6 Tenure moderation (H5)

H5, designated secondary and exploratory in the locked plan, predicted a compensatory pattern; the data pointed the other way. Condition × tenure interactions were positive and significant for self-efficacy (*b* = 0.030 per year, *p* < 0.001) and knowledge (*b* = 0.092 per year, *p* < 0.001), and absent for transfer intention (*p* = 0.49). Simple effects remained positive at −1 SD of tenure (self-efficacy *b* = 0.11, *p* = 0.041; knowledge *b* = 0.58, *p* = 0.003) but were three to four times larger at +1 SD (self-efficacy *b* = 0.49; knowledge *b* = 1.76; both *p* < 0.001), so that longer-tenured employees gained more from adaptive delivery rather than less (Figure 6). Both interactions hold under the coarser clustering levels described in the next section (company-level wild bootstrap *p* = 0.010 for self-efficacy, *p* = 0.004 for knowledge).

**Figure 6:**
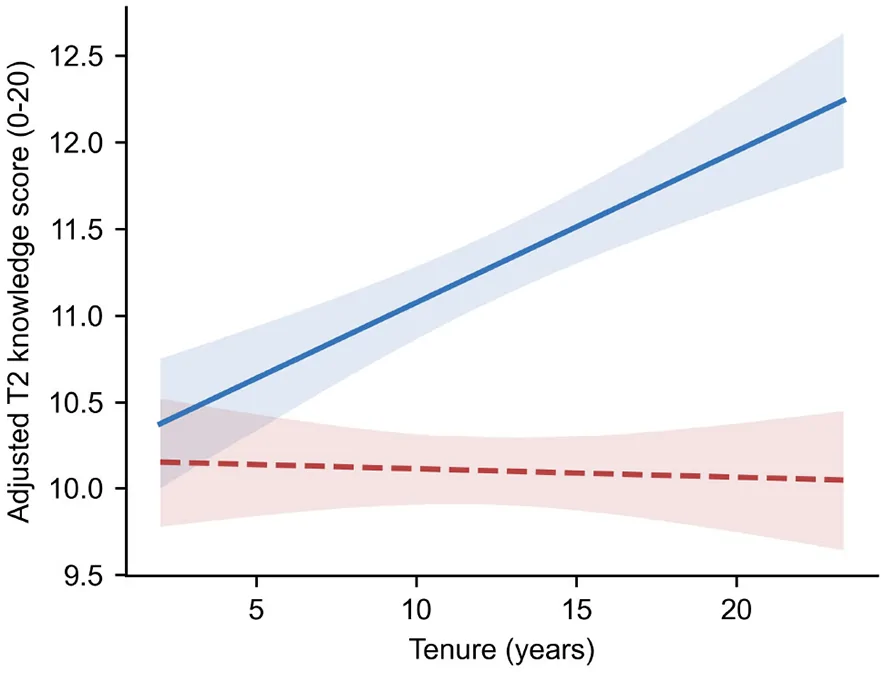
Model-adjusted T2 knowledge scores as a function of tenure by condition (solid blue, adaptive training; dashed red, traditional training), with 95% confidence bands. The adaptive advantage widens with tenure, contrary to the predicted compensatory pattern.

The interactions cannot single out tenure. When condition interactions with tenure, age, baseline knowledge, digital proficiency, and tenure^2^ were entered simultaneously (reported under Sensitivity analyses), no single linear interaction remained individually significant. These experience markers are strongly collinear (*r* = 0.85 between tenure and age) and share the moderation variance. Together with the exploratory status of H5, this provides exploratory evidence of *experience-related* heterogeneity–the advantage of adaptive delivery is larger at the more experienced end of the workforce–rather than a tenure-specific effect.

### 3.7 Robustness

Results were essentially unchanged when excluding attention-check failures (*n* = 84; e.g., knowledge *b* = 1.19, *p* < 0.001), when excluding contamination-flagged respondents (*n* = 36), under unit fixed effects with cluster-robust standard errors over the 60 batch-proxy cells, and in completer-only analyses (*b* = 1.07 for T3 knowledge). Replacing the two focal mediators with a composite load score eliminated the load pathway (all composite-load *b* paths *p*>0.10), confirming that mediation inference in this literature depends materially on respecting the multidimensional structure of cognitive load. Platform telemetry within the adaptive condition showed no significant dose–response relation between session count or path re-routing and knowledge (both *p* ≥ 0.098).

### 3.8 Sensitivity analyses

#### 3.8.1 Longitudinal mediation

Because mediators and T2 outcomes were measured concurrently, we re-estimated the parallel mediation models with T3 outcomes, temporally separating mediator (T2) from outcome (T3, controlling for T1 baselines. The pattern replicated. For T3 knowledge, both indirect effects remained significant [via extraneous load: 0.087, CI [0.026, 0.165]; via fit: 0.081, CI [0.036, 0.131]], with no pathway difference. For T3 transfer intention, the load pathway remained null [0.007, CI [−0.008, 0.025]] while the fit pathway remained significant [0.048, CI [0.023, 0.075]], and the pathway contrast remained significant [−0.041, CI [−0.072, −0.010]]: the dissociation is not an artifact of concurrent measurement. In the most conservative autoregressive specification, additionally controlling the T2 outcome, the fit pathway remained significant for both follow-up outcomes [knowledge: 0.057, CI [0.023, 0.096]; transfer intention: 0.028, CI [0.013, 0.046]] whereas the load pathways did not, and the transfer pathway contrast narrowed to marginal [−0.022, CI [−0.044, 0.000]].

#### 3.8.2 Inference without batch identifiers

Because the assignment occurred at a level we cannot observe, we did not rely on any single clustering assumption. Unit-level intraclass correlations were small (ICC = 0.006–0.027). Table 4 re-estimates every primary effect under three clustering levels, with a company-level wild cluster bootstrap, and with a stratum-level permutation test. These are alternative assumptions about the unobserved clusters rather than an ordering by conservatism: coarsening the clustering level does not mechanically enlarge standard errors, and in these data it does not always do so–the company-level standard error for T2 knowledge (0.082) is smaller than its 60-cell (0.092) and 30-unit (0.130) counterparts. Clustering at the company level treats all 500 employees of a county company as a single cluster; with only six clusters, conventional cluster-robust *p* values are unreliable, so we report Webb six-point wild cluster bootstrap-*t* *p*-values (Cameron et al., 2008; Roodman et al., 2019). The permutation test re-draws condition labels within each company × job-family unit; because the matched pairs cannot be reconstructed from the anonymized export, it approximates the allocation mechanism at the stratum level rather than reproducing it, and is reported as a sensitivity analysis rather than as design-based randomization inference. Every primary effect remains significant under every procedure; the largest *p*-value anywhere in the table is 0.008. Furthermore, design-effect corrections for hypothetical batch-level ICCs of 0.01, 0.05, and 0.10 (average batch size ≈ 40, the value implied by the approximate 65–100 batches across the 3,000 enrolled employees) left all primary effects significant under the most pessimistic scenario (largest corrected *p* = 0.0016), and individual-level propensity-score weighting reproduced the estimates (e.g., T2 knowledge *b* = 1.17).

**Table 4:** Primary condition effects under successively coarser clustering levels and two further sensitivity analyses.

		Cluster-robust SE (*p*)			Wild boot.	Permut.
Outcome	*b*	60 cells	30 units	6 companies	*p*	*p*
Training self-efficacy T2	0.297	0.028 (< 0.001)	0.040 (< 0.001)	0.038 (0.0006)	0.008	< 0.001
Transfer intention T2	0.306	0.028 (< 0.001)	0.040 (< 0.001)	0.039 (0.0005)	0.006	< 0.001
Knowledge T2	1.163	0.092 (< 0.001)	0.130 (< 0.001)	0.082 (< 0.001)	0.004	< 0.001
Knowledge T3	1.058	0.118 (< 0.001)	0.167 (< 0.001)	0.187 (0.0024)	0.008	< 0.001

All models include unit fixed effects, the T1 baseline, and the full covariate set. Coarser clustering is not necessarily more conservative: for T2 knowledge, the company-level standard error is smaller than the 60-cell and 30-unit ones. Wild bootstrap *p*-values use Webb six-point weights (2,000 replications); the permutation test re-draws condition labels within company × job-family units (2,000 permutations) and approximates, rather than reproduces, the matched-pair allocation scheme.

#### 3.8.3 Missing data

Multiple imputation (*m* = 20, chained equations with posterior draws, Rubin's rules) reproduced the available-case estimates essentially unchanged (e.g., T2 knowledge *b* = 1.17 vs. 1.15; T3 knowledge *b* = 1.05 vs. 1.07; all *p* < 0.001), and T3 dropout was jointly unpredictable from the full baseline covariate set (likelihood-ratio χ^2^(9) = 4.58, *p* = 0.87).

#### 3.8.4 Indirect-effect family FDR

Applying Benjamini–Hochberg correction to bootstrap *p*-values within the family of six indirect effects, five of six remained significant (all adjusted *p* ≤ 0.0014); only the extraneous-load pathway to transfer intention was nonsignificant (adjusted *p* = 0.73), as in the primary analysis.

#### 3.8.5 Measurement-error correction

Because the load subscales were less reliable than the fit scale (α = 0.68 vs. 0.83), differential attenuation could in principle bias the pathway comparison toward fit. Errors-in-variables re-estimation of the outcome models (error variances fixed from observed reliabilities) *strengthened* the load pathways for learning outcomes [knowledge: 0.139, CI [0.052, 0.243]; self-efficacy: 0.040, CI [0.018, 0.066]] yet left the transfer-intention dissociation intact [load pathway 0.005, CI [−0.018, 0.027]; fit pathway 0.051, CI [0.023, 0.079]; contrast −0.046, CI [−0.083, −0.010]]. The headline asymmetry therefore survives correction for unreliability.

#### 3.8.6 Competing moderators

When entering condition interactions with tenure, age, baseline knowledge, digital proficiency, and tenure^2^ simultaneously, no single linear interaction remained individually significant – tenure and age are substantially collinear (*r* = 0.85) and share the moderation variance (tenure terms retained the largest coefficients; age, baseline knowledge, and digital proficiency interactions were uniformly small, *p*≥0.29 for age on learning outcomes) — while the tenure^2^ interaction was significant for knowledge (*b* = 0.006, *p* = 0.016), indicating an accelerating advantage at longer tenure. The moderation is thus best attributed to the correlated cluster of experience variables rather than uniquely to tenure, a caveat we carry into the Discussion.

## 4 Discussion

Among 2,574 employees of six power supply companies, a knowledge-graph-driven adaptive delivery system outperformed traditional uniform training on self-efficacy, knowledge, and transfer intention. The knowledge advantage persisted 6 weeks later, with no detectable reduction over that window and any true reduction bounded below 0.10 SD by equivalence testing. The effects were small (*d* ≈ 0.21–0.29) — roughly half the magnitude of meta-analytic estimates from educational settings (Ma et al., 2014; Hillmayr et al., 2020)—but they were estimated with high precision in a real organization under real production constraints, against an active control receiving identical content. By field-intervention benchmarks, effects of this size on job-relevant knowledge and motivation across an entire workforce are practically meaningful (Kraft, 2020), and their durability at follow-up addresses the retention blind spot of the immediate-post-test literature (Baldwin and Ford, 1988).

### 4.1 Two pathways, not one—and not the same for every outcome

The mediation results provide the first parallel test of the cognitive and motivational accounts that this literature has offered for adaptive training. Both indirect pathways were statistically supported, but they served different outcomes. Learning outcomes (knowledge and the mastery-linked belief of self-efficacy) were jointly associated with reduced extraneous load and higher perceived personalization fit. Transfer intention—a motivational judgment about future behavior (Ajzen, 1991)—showed an indirect effect only through fit: a pattern consistent with employees intending to apply the training because it felt made for them rather than because it was easier to process. Because the mediators were measured rather than manipulated, these are statistical indirect effects, and mediator–outcome confounding or reciprocal influence cannot be excluded; the results are consistent with the proposed cognitive and motivational pathways but do not establish them as the operative mechanisms. Two artifactual accounts are ruled out. The dissociation replicated when mediators temporally preceded the outcome (T2 mediators to T3 transfer intention, including an autoregressive specification controlling the T2 outcome) and when mediator scores were corrected for their unequal reliabilities, so neither concurrent measurement nor differential attenuation explains it, and the confirmatory factor analysis reported in the Methods speaks against the reading that fit and transfer intention are the same construct measured twice. The pattern refines the load-centric account that dominates the adaptive-learning literature (Gkintoni et al., 2025; Halkiopoulos and Gkintoni, 2024): reduced extraneous load tracks how much people learn, whereas perceived fit tracks whether they intend to use what they learned. At the same time, the two pathways jointly accounted for only 12–19% of the total condition effects; most of the advantage remains direct and mechanistically unexplained: the field's two favored explanations appear to account for part of the observed condition effect but are far from sufficient, and identifying the residual machinery (e.g., retrieval practice embedded in adaptive delivery, pacing autonomy) is a concrete agenda for future work. Organizations optimizing only for processing efficiency may therefore improve test scores without moving the motivational outcomes that determine whether training reaches the job.

The load-subscale pattern carries its own theoretical message. We expected intrinsic load to fall alongside extraneous load; instead, adaptive training reduced extraneous load, as designed, but simultaneously pushed learners into more challenging content—visible as elevated intrinsic load—and this elevation partially suppressed the benefits. This interpretation is *post-hoc*, but it is consistent with evidence that adaptivity does not minimize load so much as redistribute it (Mo et al., 2022; Zeitlhofer et al., 2024), and with the long-standing argument that the load types are functionally distinct and should not be aggregated (Sweller, 2010; Kalyuga, 2011). It also explains why our composite-load robustness check found nothing: the subscales moved in opposite directions and canceled. Studies that measure cognitive load as a single score (cf. Leppink et al., 2013; Zhu et al., 2024) may be systematically underestimating—or simply missing—mediation that is present at the subscale level.

### 4.2 Experience-related heterogeneity where a compensatory pattern was predicted

We predicted, following Brynjolfsson et al. (2023) and Walkington (2013), that adaptive training would help inexperienced employees most. The exploratory moderation analysis pointed the other way: benefits for knowledge and self-efficacy grew with tenure, and the interactions held up under company-level wild bootstrap inference. Of the two theoretical accounts we set against each other a priori, these data are more consistent with the expertise reversal account (Kalyuga, 2007): in generative-AI field studies, novices receive direct performance scaffolding for tasks they cannot yet do, whereas here the platform personalizes *learning* of material for which longer-tenured employees possess richer schemas to which new content can be attached—and adaptive sequencing is precisely the mechanism that lets experienced workers skip the redundant basics that uniform training forces on them, the signature condition under which learner-tailored instruction favors experts. Two attribution caveats apply. When correlated experience-related moderators entered the model jointly, the moderation variance was shared between tenure and age, with a significant accelerating tenure^2^ term for knowledge, so what the data show is that the experienced end of the workforce benefits more, not that tenure specifically is the active moderator—a pattern for confirmatory test in future work rather than a tenure-specific Matthew effect. And the condition × baseline-knowledge interaction was null, which complicates a purely schema-based reading: whatever experienced workers leverage is not captured by their score on our 20-item knowledge pretest; accumulated procedural and contextual job knowledge, which the test does not measure, is the more plausible substrate. Relatedly, how workers engage with AI systems is not uniform: recent work distinguishes dependent from autonomous cognitive offloading to generative AI, with different consequences for the user (Zhu et al., 2026)—veterans may be better positioned to engage adaptively autonomously, treating the platform as a tool for directing their own learning rather than deferring to it. Whatever the mechanism, the pattern matters for deployment policy: contrary to the intuitive strategy of targeting adaptive platforms at new hires, the immediate returns in this setting were largest among veterans, while shorter-tenured employees still benefited (simple effects remained positive at −1 SD). The finding also underscores that aptitude—treatment interactions in workplace AI cannot be assumed from educational or task-assistance contexts (Poupard et al., 2024).

### 4.3 Practical implications

For utilities and comparable high-reliability industries, three implications follow. First, the platform-based adaptive package as a whole—not adaptivity in isolation—outperformed the standard uniform refresher on job-relevant knowledge, and did so against a content-matched active control at workforce scale, making it a reasonable candidate for adoption when compared with conventional refresher delivery. That case rests on knowledge and on self-reported motivation: we measured intention to transfer rather than transfer behavior, and collected no data on job performance, safety outcomes, error rates, or the cost of platform deployment relative to classroom delivery, so claims about returns on those dimensions would go beyond this evidence. Second, platform design should treat perceived fit as a first-class design target—surfacing to employees *that* and *how* the system is adapting to them—since fit rather than ease was the pathway associated with transfer intention; this converges with evidence that personalization unnoticed by learners fails to move outcomes (Lim et al., 2022; Bartolomé et al., 2018). Third, and as a *post-hoc* reading of the load-subscale pattern, adaptive difficulty progression may need tuning to keep the intrinsic-load elevation productive rather than discouraging, since that elevation carried a small negative indirect effect on self-efficacy.

### 4.4 Limitations

Six limitations bound these conclusions. First, the contrast is between a bundled adaptive delivery system and standard uniform training. The two arms were matched on syllabus, calendar window, scheduled hours, assessment standard, and instructor pool (Table 1), but adaptive delivery necessarily brings a different medium (platform-based rather than instructor-led), reduced synchronous instructor contact, self-pacing, an entry diagnostic, item-level feedback, and more frequent embedded retrieval practice, whose effect on retention is well documented (Roediger and Karpicke, 2006). The estimates identify the effect of the package, not of adaptivity or of AI as such; isolating components would require a factorial design—adaptive vs. fixed sequencing within the same platform, or platform delivery with and without adaptive routing—that this field implementation could not accommodate.

Second, allocation was randomized within matched pairs of batches, but batch identifiers were not carried into the anonymized export, with two consequences. The randomization cannot be audited from the analysis dataset—the allocation ledger documents it, the data cannot corroborate it—and no analysis can condition on, or cluster at, the level at which treatment was actually assigned. Baseline balance was excellent and the effects held under clustering at three levels, a company-level wild bootstrap, and a stratum-level permutation test (Table 4); but each of these approximates inference at the true assignment level rather than performing it, and residual cluster-level imbalance therefore cannot be ruled out empirically.

Third, the outcomes were knowledge, self-efficacy, and transfer *intention* over 6 weeks in one provincial utility. Whether intention converts into transfer behavior is not automatic (Blume et al., 2010), and job performance, safety outcomes, and cost-effectiveness lay outside the measurement agreement with the host organization. Generalization to other industries and to workforces with different tenure and digital-literacy profiles awaits replication, as does cross-validation of the two purpose-built scales beyond the structure and invariance established here.

Fourth, the novelty of the adaptive platform is an alternative-explanation risk this design cannot fully exclude: employees may have responded in part to the newness of the delivery format itself rather than to adaptive sequencing or personalization. The undiminished T3 advantage established by equivalence testing and the absence of a dose–response relation between platform exposure and knowledge within the adaptive arm sit awkwardly with a pure novelty or Hawthorne-type account, which would predict decay with repeated exposure, but neither observation rules the explanation out.

Fifth, the analysis plan was locked internally before data collection but was not deposited in a public registry; the plan and every departure from it are documented in Supplementary material S4, so temporal precedence rests on our internal record rather than on third-party verifiable timestamping.

Sixth, perceived personalization fit plays a dual role in this design: it is both the candidate mediator specified in H3 and, in effect, a check that the adaptive manipulation was subjectively experienced as personalized—a point Reviewer 1 asked us to make explicit. Because the same six-item measure serves both functions, the mediation and manipulation-check readings cannot be fully disentangled statistically; a null or weak effect on this measure would be ambiguous between “personalization did not help” and “personalization was not perceived as such.”

### 4.5 Conclusion

In a large three-wave cluster-randomized field experiment inside a power utility, a knowledge-graph-driven adaptive delivery system produced small and robust advantages over uniform training in knowledge, self-efficacy, and transfer intention, with no detectable erosion of the knowledge advantage over 6 weeks. The advantages were consistent with two distinct pathways—lighter extraneous processing and a stronger sense that the training fit the person—with the motivational outcome linked to fit alone, and exploratory analyses suggest they accrued disproportionately to more experienced employees. In this organization, adaptive workplace training worked—but not only, and not always, for the reasons the literature has assumed.

## Data Availability

The de-identified dataset and analysis code are not publicly deposited, in keeping with the datasharing agreement with the host organization; they are available from the corresponding author upon reasonable request, subject to the host organization's data-governance approval, for the purpose of verifying the analyses reported here.

## Supplementary material

Supplementary Material S1 Assignment mechanism, allocation rule and ledger template, and batch structure.

Supplementary Material S2 Full item content of all self-report measures in English and Chinese, with facet mapping and scoring rules.

Supplementary Material S3 Confirmatory factor analysis, reliability, discriminant validity and measurement invariance across conditions, including the sensitivity analysis for the weakly loading self-efficacy item (S3.6).

Supplementary Material S4 Locked analysis plan, complete model specifications, multiplicity handling, and deviation log.

Supplementary Material S5 Analysis code and output archive.
