# Supplementary material for: AI-driven adaptive training for utility employees: a three-wave cluster-randomized field experiment on cognitive-load and personalization-fit pathways
Source: Front Psychol. 2026 Sep 17;17:1944626. doi: 10.3389/fpsyg.2026.1944626 (PMC13626984; doi:10.3389/fpsyg.2026.1944626)
Supplement: Supplementary file 1 [file Data_Sheet_1.pdf]

Supplementary Material

AI-driven adaptive training for utility employees: a three-wave cluster-randomized field experiment on cognitive-load and personalization-fit pathways

Contents

- S1. Assignment mechanism, allocation ledger, and batch structure
- S2. Full item content of all self-report measures
- S3. Confirmatory factor analysis, reliability, and measurement invariance
- S4. Locked analysis plan, complete model specifications, and deviation log
- S5. Analysis code and output archive

S1. Assignment mechanism, allocation ledger, and batch structure

S1.1 Allocation rule (fixed before enrollment)

1. The HR department of the host utility supplied the complete roster of training batches scheduled for the annual technical-refresher curriculum in the six participating county-level companies. For each batch the roster recorded: batch size, company, job family, mean tenure, mean prior-training score, and prior-training participation count.
2. Within each company and job family, batches were sorted on these batch-level characteristics and formed into matched pairs, pairing batches that were closest on mean tenure and prior-training record.
3. Within each matched pair, the research team drew a random number to allocate one batch to the adaptive condition and the other to the traditional condition.
4. Allocation was performed by the research team, not by line managers, the training vendor, or the platform provider. No batch characteristic —performance history, supervisor preference, site, or scheduling convenience —was permitted to influence which arm a batch entered.
5. The allocation was fixed and entered into a written ledger before T1 measurement and before any employee was informed of their training format.
6. Any batch that could not be allocated by the matched-pair draw for operational reasons (e.g. equipment constraints requiring on-site delivery) was to be recorded in the ledger as a non-random assignment, with the reason stated.

S1.2 Allocation ledger fields

Field	Content
Batch ID	Internal HR identifier
Company	One of six county-level companies
Job family	Engineering / operations and maintenance / marketing / support / other
Batch size	Number of employees scheduled
Matching pair ID	Identifier of the matched pair
Mean tenure, mean prior-training score	Batch-level matching variables
Allocated condition	Adaptive / traditional
Allocation method	Random draw within matched pair / non-random (reason recorded)
Date of allocation	Recorded before T1

S1.3 Batch structure

The study was embedded in the host organization's existing annual training programme rather than mounted as a purpose-built experiment; dedicated research batches, purpose-built classes, and recruitment into a separate protocol were not available options inside an operating utility. Batch structure therefore reflects the training department's operational constraints throughout.

Batch size was determined by the host organization's facilities rather than by the research design. Training rooms differed substantially in capacity across the six county companies —from roughly a dozen places at the smallest sites to about 100 at the largest —so batches were markedly unequal in size. Across the 3,000 enrolled employees this places the total at approximately 65–100 batches, i.e. roughly five to eight per condition per company.

Two separate facts should be distinguished, because they have different implications.

At allocation, batches were well defined. The ledger listed each scheduled batch as a row, matched pairs were formed among those rows within company and job family, and the random draw was made within each pair. The allocation mechanism therefore operated on a definite set of units.

At implementation, the batch ceased to be a stable unit. Room capacity and schedule changes led to batches being merged and split *within condition*, so the groups that actually attended a session together no longer correspond one to one with the batches recorded at allocation. Merging across conditions could not occur, because the two arms were delivered in different formats —platform-based individual sessions versus instructor-led classroom sessions —and never shared a session; the contamination probe administered at T2 is consistent with this (1.4% affirmative).

For this reason batch identifiers were not carried into the anonymized data export supplied to the research team: after these operational changes they no longer identified the units on which allocation had been performed. Exact batch membership therefore cannot be reconstructed at the individual level.

The ledger itself is held by the host organization's HR department, the anonymized export being the only data transferred under the data-sharing agreement. It was an operational document, archived through the organization's routine records process once the training cycle closed rather than maintained as research documentation. The delivered sessions are not undocumented —attendance registers and facility scheduling records exist at each site —but assembling a session-by-session reconstruction from them would require retrieving and manually collating paper and archived administrative records for approximately 3,000 employees across six separate county companies, and would recover the groups that attended together rather than the batches on which allocation was performed. We judge that effort disproportionate to what it would add. Should the editorial office require it, we will request a batch-level summary of the ledger —counts and condition assignments only, containing no individual-level information —and supply whatever can be retrieved.

The observable strata within which batches were formed are:

Level	Number	Size (M)	Size range
Company	6	429	—
Company × job family (unit)	30	85.8	39–152
Company × job family × condition (cell)	60	42.9	16–76

Section S4.4 and Table 4 of the main text report inference under three successively coarser clustering levels, a company-level wild cluster bootstrap, and a stratum-level permutation test. Because the matched-pair structure cannot be reconstructed from the anonymized export, that permutation test re-draws condition labels within company × job-family strata and therefore approximates, rather than exactly reproduces, the matched-pair scheme by which batches were allocated.

S2. Full item content of all self-report measures

All self-report items were administered in Chinese. English renderings below are provided for review; they were not used in data collection. Unless noted, items used 7-point Likert scales (1 = *strongly disagree*, 7 = *strongly agree*), and composites are item means.

S2.1 Perceived personalization fit (PFP; T2; 6 items; purpose-built)

Construct definition. An employee's subjective judgment that the training's content difficulty, job relevance, learning path, pace, and responsiveness match their own level, post requirements, and learning needs.

Development. (a) The construct definition was derived from personalization research (Walkington, 2013) and task–technology fit logic (Goodhue & Thompson, 1995). (b) The definition was decomposed into six content facets. (c) One item was written per facet and worded for the electric-power training context. (d) Items were reviewed by the curriculum experts

who authored the training syllabus for comprehensibility and contextual appropriateness. (e) Items make no reference to any platform or to adaptivity, so that employees in both conditions answer on identical terms. (f) The questionnaire section was labelled "training experience"rather than "personalization"to avoid cueing the study hypothesis.

Item	Facet	English	Chinese
PFP1	Level match	The difficulty and pace of this training matched my current level of knowledge.	本次培训的内容难度和进度与我目前的知识水平是匹配的。
PFP2	Job-requirement match	The cases and situations presented in the training were highly relevant to the requirements of my own job.	培训中呈现的案例或情境与我实际工作岗位的需求高度相关。
PFP3	Recognition of weak points	The training was able to identify my individual weak points and provide content targeted at them.	培训系统或讲师能够识别出我个人的薄弱环节，并针对性地提供内容。
PFP4	Overall customization	This training felt tailor-made for me rather than one-size-fits-all.	我感觉这次培训是"为我量身定制的"，而不是"一刀切"的统一内容。
PFP5	Path and pace match	The learning path and sequence of the training suited my own learning pace and preferences.	培训的学习路径或顺序符合我个人的学习节奏和偏好。
PFP6	Responsive support	When I had difficulty with a knowledge point, the training provided additional help matched to what I needed.	如果我在某个知识点上遇到困难，培训会提供与我需求匹配的额外帮助。

All items are positively keyed; the composite is the mean of the six items. Cronbach's α = .83; composite reliability = .84.

S2.2 Training self-efficacy (TSE; T1 and T2; 7 items; adapted)

Development. Adapted to the training domain from the general self-efficacy template (Zhang & Schwarzer, 1995; Bandura, 1977). Items were written to span mastery experience, outcome expectancy, generalization to future learning, and autonomous problem solving. Item 7 is reverse-keyed to control acquiescence.

Item	English	Chinese
TSE1	Through this training, I am confident that I can master the core knowledge and skills it covers.	通过这次培训，我相信自己能够掌握本次培训涉及的核心知识/技能。
TSE2	Even when the training content is difficult, I am confident I can understand and master it through effort.	即使遇到较难的培训内容，我也有信心通过努力理解并掌握。
TSE3	I am confident that I can correctly apply what I learned in this training in my actual work.	我相信自己能够在实际工作中正确运用本次培训所学的内容。
TSE4	Compared with before the training, I now feel more confident about completing related work tasks.	与培训前相比，我现在对完成相关工作任务更有信心。

Item	English	Chinese
TSE5	I believe my learning ability is sufficient for future training or new-technology learning of a similar kind.	我相信自己的学习能力足以应对未来类似的培训/新技术学习任务。
TSE6	Even without guidance from others, I am confident I can solve problems I encounter in the training content.	即使没有他人指导，我也有信心自己解决培训内容中遇到的问题。
TSE7 (R)	Faced with the content of this training, I often worry that I will not be able to learn it.	面对这次培训的内容，我常常担心自己学不会。

Item 7 is reverse-scored before averaging. Cronbach's α = .82 at both waves; composite reliability = .83.

S2.3 Transfer intention (TTI; T1, T2, T3; 6 items; adapted)

Development. Written within the theory-of-planned-behaviour framework (Ajzen, 1991) and the training-transfer tradition (Baldwin & Ford, 1988), covering behavioural intention, attitude toward applying the content, intention strength, perceived support, and overall intention.

Item	English	Chinese
TTI1	I intend to try to apply the knowledge and methods learned in this training in my actual work.	我打算在实际工作中尝试运用本次培训所学的知识/方法。
TTI2	I believe that applying the training content to my work will help my job performance.	我相信将培训内容应用到工作中会对我的工作表现有帮助。
TTI3	I plan to actively look for opportunities to apply this training's content within the next month.	我计划在接下来的一个月内主动寻找机会应用本次培训的内容。
TTI4	Even when work is busy, I will make time to try to apply what I learned.	即使工作繁忙，我也会优先安排时间尝试运用培训所学。
TTI5	I think the atmosphere in my team or unit supports my applying the training content in actual work.	我认为班组/单位的氛围支持我把培训内容用到实际工作中。
TTI6	Overall, I have a strong intention to turn this training into actual work behaviour.	总体而言，我有很强的意愿把这次培训转化为实际工作行为。

Cronbach's α = .82–.83; composite reliability = .84.

S2.4 Cognitive load (T2; 10 items; published instrument)

Ten items from the three-factor instrument of Leppink et al. (2013), administered in the validated Chinese version (MDT-CL; Zhang et al., 2020, *Journal of Nursing Management*, 28, 277–285), rated 0–10. Item content is not reproduced here for copyright reasons; the assignment of items to factors follows the published instrument: intrinsic load (3 items), extraneous load (3 items), germane load (4 items). Cronbach's α in this sample = .68 / .68 / .73; composite reliability = .69 / .69 / .73.

S2.5 Knowledge test (T1, T2, T3; 20 items per form)

Three parallel forms constructed by the curriculum experts from a common blueprint: identical module weighting, identical item formats (single-choice and true/false), identical target difficulty band, automatic objective scoring (0–20). Item content is not reproduced because the forms remain in operational use by the host organization. Form statistics are reported in the main text (Measures) and in S3.4.

S2.6 Covariates and quality-control items

Digital-device proficiency (3 self-report items, 7-point), tenure in years, education, prior training participation count, and the corresponding T1 baseline of each outcome. The T2 questionnaire embedded one instructed-response attention check and one contamination probe ("Have you discussed the content or format of this training with colleagues trained in the other format?").

S3. Confirmatory factor analysis, reliability, and measurement invariance

Estimator: MLR (robust maximum likelihood). Software: R 4.x, lavaan 0.6. Sample: n = 2,318 employees with complete T2 self-report data (1,156 adaptive, 1,162 traditional).

S3.1 Model fit

Model	χ^2 (scaled)	df	CFI	TLI	RMSEA	SRMR
Six-factor (hypothesized)	411.3	362	.997	.997	.008	.018
Five-factor (fit and transfer intention merged)	3,956.2	367	.810	.790	.065	.064
One-factor (Harman extreme comparison)	10,979.9	377	.437	.394	.111	.108

The five-factor comparison addresses the concern that perceived personalization fit and transfer intention may be semantically redundant: merging them costs ΔCFI = −.187. The one-factor comparison addresses pervasive common-method variance.

S3.2 Standardized loadings

Factor	Item	Std. loading	SE	z
Intrinsic load	CL_I_01	.757	.021	36.0
Intrinsic load	CL_I_02	.660	.021	31.5
Intrinsic load	CL_I_03	.537	.020	26.6
Extraneous load	CL_E_01	.779	.022	35.9
Extraneous load	CL_E_02	.668	.021	32.6
Extraneous load	CL_E_03	.509	.021	24.8
Germane load	CL_G_01–04	.49–.74	≤ .022	all p < .001
Perceived personalization fit	PFP_01–06	.54–.77	≤ .022	all p < .001
Training self-efficacy	TSE_01–07	.24–.78	≤ .023	all p < .001
Transfer intention	TTI_01–06	.43–.79	≤ .022	all p < .001

The complete item-by-item table with all standard errors, z values, and p values is provided in the output archive (`results/cfa_loadings.md`). The lowest loading (.24) is **TSE7**, the single reverse-keyed self-efficacy item ("Faced with the content of this training, I often worry that I will not be able to learn it"/ 面对这次培训的内容，我常常担心自己学不会), a common method effect of reverse keying. Section S3.6 reports the full comparison of the measurement model and of every substantive result with and without this item.

S3.3 Reliability, AVE, and factor correlations

Factor	Items	Loading range	CR	AVE	√AVE
Intrinsic load	3	.54−.76	.692	.432	.657
Extraneous load	3	.51−.78	.694	.437	.661
Germane load	4	.49−.74	.732	.411	.641
Perceived personalization fit	6	.54−.77	.836	.462	.680
Training self-efficacy	7	.24−.78	.825	.420	.648
Transfer intention	6	.43−.79	.835	.465	.682

AVE values fall between .41 and .47, below the conventional .50 benchmark; with composite reliabilities above .70 this is generally judged acceptable convergent validity (Fornell & Larcker, 1981).

Factor correlations (standardized): the largest is .416 (self-efficacy with transfer intention), followed by .352 (fit with transfer intention) and .348 (fit with self-efficacy). All remaining correlations are below .23. Every correlation is smaller than the smallest √AVE (.641), so the Fornell−Larcker criterion is satisfied for every pair. Heterotrait−monotrait ratios: fit with transfer intention .35, fit with self-efficacy .33, fit with extraneous load .02 (threshold .85).

S3.4 Measurement invariance across conditions

Model	χ^2 (scaled)	df	CFI	RMSEA	SRMR	ΔCFI	ΔRMSEA
Configural	810.5	724	.995	.010	.024	—	—
Metric (equal loadings)	825.3	747	.996	.010	.024	+.000	−.001
Scalar (equal loadings and intercepts)	845.9	770	.996	.009	.025	+.000	−.000

Scalar invariance holds, well within the $|\Delta CFI| < .010$ convention (Cheung & Rensvold, 2002). Between-condition mean comparisons and the *b* paths in the mediation models are therefore made on a common measurement metric.

S3.5 Knowledge-test form equivalence

Form	n	Item difficulty M (SD)	Difficulty range	Mean point-biserial	KR-20	Total M (SD)
T1	2,525	.509 (.183)	.24−.79	.350	.789	10.17 (4.17)
T2	2,428	.534 (.179)	.26−.84	.340	.779	10.68 (4.10)
T3	2,359	.547 (.178)	.26−.84	.351	.789	10.93 (4.17)

Difficulty distributions do not differ between the T1 and T2 forms, $t(38) = -0.44$, $p = .66$, or between the T2 and T3 forms, $t(38) = -0.22$, $p = .83$. Both conditions took the same form at each wave, so between-condition contrasts are unaffected by any residual form difference.

S3.6 Sensitivity to the weakly loading self-efficacy item (TSE7)

Added post hoc in response to peer review, round 2.

TSE7 is the one reverse-keyed item of the training self-efficacy scale. Its standardized loading is .242 and its corrected item − total correlation with the remaining six items is .224. Below, the seven-item composite (the pre-specified scoring, retained in the manuscript) is compared with a six-item composite that drops TSE7, on the measurement model and on every substantive result in which self-efficacy appears. Script: `p4_tse_item_sensitivity.py`; output: `results/p4_tse_item_sensitivity.md`.

Measurement.

Index	7 items (reported)	6 items (TSE7 dropped)
Six-factor CFA, χ^2 (scaled) / df	411.3 / 362	343.0 / 335
CFI / TLI	.997 / .997	1.000 / 1.000
RMSEA / SRMR	.008 / .018	.003 / .016
TSE loading range	.24–.78	.60–.78
TSE composite reliability	.825	.846
TSE average variance extracted	.420	.480
Cronbach α, T1 / T2	.816 / .815	.838 / .843
Configural / metric / scalar invariance across conditions	supported	supported (CFI .998 throughout; RMSEA .008 / .007 / .006)

Dropping the item improves every index. The two composites correlate $r = .980$ at T2.

Substantive results.

Result	7 items (reported)	6 items (TSE7 dropped)
H1a condition effect on T2 self-efficacy (LMM)	$b = 0.297$, SE = 0.038, $p < .001$, $d = 0.26$	$b = 0.307$, SE = 0.041, $p < .001$, $d = 0.25$
Cluster-robust SE, 60 cells / 30 units / 6 companies	.028 / .040 / .038 (all $p < .001$)	.029 / .041 / .048 (all $p < .001$)
Indirect effect via extraneous load [95% CI]	0.026 [0.012, 0.043]	0.027 [0.010, 0.046]
Indirect effect via perceived fit [95% CI]	0.029 [0.015, 0.045]	0.034 [0.017, 0.051]
Pathway contrast [95% CI]	−0.003 [−0.023, 0.017]	−0.007 [−0.031, 0.017]
Direct effect c'	0.238	0.243
H5 condition × tenure interaction	$b = 0.030$ per year, $p < .001$	$b = 0.032$ per year, $p < .001$
Simple slope at −1 SD tenure	0.108, $p = .041$	0.105, $p = .069$
Simple slope at +1 SD tenure	0.485, $p < .001$	0.509, $p < .001$

Every conclusion is unchanged: the main effect is if anything slightly larger, both indirect effects remain significant with no pathway difference, and the condition × tenure interaction retains its sign, magnitude, and significance. The one numerical difference worth flagging is the simple slope at −1 SD of tenure, which moves from $p = .041$ to $p = .069$ while its point estimate is essentially identical (0.108 vs 0.105); this does not bear on the reported conclusion, which is that the advantage is larger at the more experienced end of the workforce.

S4. Locked analysis plan, complete model specifications, and deviation log

S4.1 Status of the plan

The analysis plan was specified and internally locked before data collection. It fixed: the hypotheses and their primary/secondary status, the outcome and mediator variables, the covariate set, the exclusion rules, the clustering approach, the multiplicity correction, and the mediation estimator. It was not lodged with a third-party registry.

S4.2 Complete model specifications

H1, single-time-point outcomes (T2 self-efficacy, T2 transfer intention).

```
Y_i = β0 + β1 · Condition_i + β2 · Y_i,T1 + β3 · DigitalProficiency_i + β4 · Tenure_i
      + β5 · Education_i + β6 · PriorTrainingCount_i + u_j[i] + ε_i
```

where u_j is a random intercept for the company × job-family unit (30 units), REML estimation.

H1b, knowledge retention (repeated measures over T2 and T3).

```
Y_it = β0 + β1 · Condition_i + β2 · Time_t + β3 · (Condition_i × Time_t) + β4 · Knowledge_i,T1
       + β5 · DigitalProficiency_i + β6 · Tenure_i + β7 · Education_i + β8 · PriorTrainingCount_i
       + Σ δ_u · Unit_u,i + v_i + ε_it
```

with individual random intercept v_i and unit fixed effects; T3 simple effect estimated in a separate model on T3 knowledge with the same covariate set and a unit random intercept.

H2–H4, parallel mediation. Three OLS equations per outcome, estimated on the same analysis sample and bootstrapped jointly:

```
CL–E_i  = a01 + a1 · Condition_i + covariates + e1
PFP_i   = a02 + a2 · Condition_i + covariates + e2
Y_i     = c0 + c' · Condition_i + b1 · CL–E_i + b2 · PFP_i + covariates + e3
```

Indirect effects $a1 \cdot b1$ and $a2 \cdot b2$; H4 contrast = $a1 \cdot b1 - a2 \cdot b2$. Inference: 5,000 cluster-bootstrap resamples over the 60 company × job-family × condition cells, percentile intervals; resamples in which only one condition appears are redrawn.

H5, moderation. H1 models plus Condition × Tenure (tenure mean-centred), with simple slopes at ±1 SD computed from the coefficient covariance matrix.

S4.3 Multiplicity

Benjamini–Hochberg correction was applied within the family of H1–H3 primary tests, and separately within the family of six indirect effects (three outcomes × two mediators). This procedure bounds the false discovery rate, not the familywise error rate.

S4.4 Deviation log

Every analysis in the manuscript is classified below. Analyses marked *post hoc* were not in the locked plan and are labelled as such at first mention in the text.

Analysis	Status	Trigger
H1 main effects, mixed models	Pre-specified	Locked plan
H1b repeated-measures knowledge model and T3 simple effect	Pre-specified	Locked plan
H2–H3 parallel mediation, cluster bootstrap	Pre-specified	Locked plan
H4 pathway contrast	Pre-specified (non-directional, exploratory)	Locked plan
H5 tenure moderation	Pre-specified as secondary/exploratory	Locked plan
Exploratory decomposition across all three load subscales	Pre-specified as exploratory	Locked plan
Robustness: attention-check and contamination exclusions, fixed vs random effects, completers only, composite-load respecification	Pre-specified	Locked plan
Longitudinal mediation (T2 mediators → T3 outcomes) and autoregressive variant	Post hoc	Internal review, round 1
Multiple imputation (m = 20)	Post hoc	Internal review, round 1
FDR within the family of six indirect effects	Post hoc	Internal review, round 1
Errors-in-variables correction of *b* paths	Post hoc	Internal review, round 1
Design-effect corrections for hypothetical batch ICCs	Post hoc	Internal review, round 1
Propensity-score weighting	Post hoc	Internal review, round 1

Analysis	Status	Trigger
Confirmatory factor analysis, competing models, measurement invariance	Post hoc	Peer review, round 1
Equivalence (TOST) and non-inferiority tests of durability, **including the choice of the 0.10 SD smallest effect size of interest and of the 50% non-inferiority margin**	Post hoc	Peer review, round 1
Cluster-robust SEs at three levels; Webb wild cluster bootstrap; stratum-level permutation test	Post hoc	Peer review, round 1
Knowledge-form difficulty, discrimination, and KR-20 comparison	Post hoc	Peer review, round 1
Within-condition pre–post change tests	Post hoc	Peer review, round 1
Competing-moderator model (tenure, age, baseline knowledge, digital proficiency, $tenure^2$)	Post hoc	Internal review, round 1
Sensitivity of the measurement model and of all self-efficacy results to dropping TSE7 (S3.6)	Post hoc	Peer review, round 2

No hypothesis, outcome, or exclusion rule was changed after data collection. No analysis was removed from the locked plan.

Note on the durability criteria. The locked plan specified the condition × time interaction as the test of H1b's durability claim, and nothing else. Both the 0.10 SD smallest effect size of interest and the criterion that the T3 effect retain at least half of the T2 effect were selected after the first round of peer review and are therefore post hoc. Their substantive justification is stated in the Methods: 0.10 SD of the knowledge score is 0.42 points, less than the value of a single item on a 20-item test, so a difference below that bound is not interpretable as decay for a training-evaluation decision; the halving criterion is the conventional boundary between substantial retention and marked erosion, was defined as a proportion of the estimated T2 effect, and was fixed before the T3 estimate was inspected. Neither criterion was adjusted after seeing the equivalence or non-inferiority results.

S5. Analysis code and output archive

File	Content
`cleaning.py`	Construction of the analysis dataset from the raw export, exclusion rules, composite scoring
`analysis.py`	Primary analyses: descriptives, baseline balance, reliability, H1–H5, FDR, figures
`p1_analysis.py`	Longitudinal mediation, multiple imputation, indirect-effect FDR, errors-in-variables correction, competing moderators
`p2_analysis.py`	Design-effect scenarios, propensity-score weighting, further robustness checks

File	Content
`p3_analysis.py`	Participant flow, implementation-fidelity indicators, batch structure, three-level clustering, wild cluster bootstrap, stratum-level permutation test, equivalence and non-inferiority tests, knowledge-form statistics, within-condition change
`p4_tse_item_sensitivity.py`	Re-estimation of the measurement model, main effect, clustering, mediation, and moderation with TSE7 dropped (S3.6)
`cfa.R`, `cfa_loadings.R`	Confirmatory factor analysis, competing models, reliability and AVE, measurement invariance, item-level loadings
`results/*.md`	Complete numerical output of every analysis reported in the manuscript

Software: Python 3.11 (statsmodels 0.14, scipy 1.15, pandas, numpy), R 4.x (lavaan 0.6). Random seeds are fixed in each script.

The de-identified analysis dataset is available from the corresponding author under the data-sharing terms agreed with the host organization, which permit sharing for verification of the reported analyses.
